# The role of histone modifications in transcription regulation upon DNA damage

**DOI:** 10.1002/1873-3468.70241

**Published:** 2025-11-28

**Authors:** Angelina Job Kolady, Siyao Wang

**Affiliations:** ^1^ Institute for Genome Stability in Ageing and Disease, Medical Faculty University of Cologne Germany; ^2^ Institute of Molecular Biology Mainz Germany

**Keywords:** acetylation, DNA damage response, DNA repair, genome stability, histone modification, methylation, PARylation, phosphorylation, recovery, repression, transcription, ubiquitination

## Abstract

Cells are constantly exposed to various sources of DNA damage, including radiation, chemicals, replicative stress and oxidative stress, that threaten genome stability. To ensure faithful DNA repair, transcription regulation needs to be tightly controlled. This regulation involves transcriptional suppression, selective activation of DNA repair‐related genes and transcriptional recovery post‐repair. Failure to properly modulate transcription during DNA damage can result in collisions between transcriptional and repair machineries, misregulation of repair genes and delayed recovery, ultimately compromising genomic integrity. Chromatin modifications play a central role in this process. These modifications include phosphorylation, methylation, acetylation and ubiquitination, which orchestrate DNA accessibility for repair machinery and fine‐tune transcriptional responses. Absence of these modifications leads to inefficient DNA repair and transcriptional errors that are implicated in diseases such as cancer, premature ageing and neurodegenerative disorders. In this review, we delve into the role of various types of histone modifications, such as phosphorylation, methylation, acetylation and ubiquitination and how they regulate transcription in response to DNA damage.

**Impact Statement**

This review elucidates how histone modifications orchestrate transcription regulation during DNA damage response, safeguarding genome stability. We also discuss transcription dysregulation in diseases such as cancer and premature aging. Our review provide insights on chromatin‐based repair pathways and guide researchers in developing therapeutic targets.

## Graphical abstract

This review discusses the critical role of histone modifications in regulating gene expression during the DNA damage response (DDR). By modulating chromatin structure and recruiting repair factors, these post‐translational modifications fine‐tune transcriptional programmes to maintain genomic stability. Understanding these mechanisms provides insights into diseases like cancer and highlights potential therapeutic targets. Created in biorender. Wang, S. (2026) https://BioRender.com/hddjovi

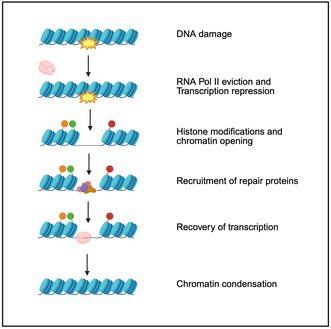

## Abbreviations

**ART**, ADP‐ ribosyltransferase

**ATM**, ataxia‐telangiectasia‐mutated

**ATP**, adenosine tri phosphate

**ATR**, ataxia – telangiectasia and *Rad3‐related*

**BER**, base excision repair

**BRCT**, BRCA 1 terminus

**BRD4**, *bromodomain‐containing protein 4*

**CAT**, catalytic domain

**CDK**, cyclin‐dependent kinase

**CS**, Cockayne syndrome

**CTD**, C‐ terminal domain

**DDR**, DNA damage response

**DNA**, deoxyribonucleic acid

**DOT1L**, disruptor of telomeric silencing 1‐like

**DPC**, DNA–protein crosslink

**DSB**, double‐stranded break

**DSIF**, DRB sensitivity‐inducing factor

**ERK**, extracellular signal‐regulated kinase

**EZH2**, enhancer of zeste homologue 2

**FACT**, facilitating chromatin

**HAT**, histone acetyl transferase

**HD**, helical domain

**HDAC**, histone deacetylase

**KAP 1**, *Krüppel‐associated box domain‐associated protein‐1*

**MDC1**, mediator of DNA damage checkpoint protein 1

**MRE11**, meiotic recombination 1

**NBS1**, Nijimegen breakage syndrome 1

**NELF**, negative elongation factor

**NER**, nucleotide excision repair

**NHEJ**, non‐homologous end joining

**NuRD**, nucleosome remodelling and deacetylase

**PARP**, poly ADP Ribose polymerase

**PcG**, polycomb group

**PD**, Parkinson's disease

**PIC**, pre‐initiation complex

**PKA**, protein kinase A

**PRC**, polycomb repressive complex

**P‐TEFb**, positive transcription elongation factorb

**PTM**, post‐translational modification

**RNA Pol II**, RNA polymerase II

**SAM**, S‐adenosyl‐methionine

**SET 1D**, SET domain containing 1A

**SSB**, single‐stranded break

**TBL**, transcription‐blocking lesion

**TBP**, TATA–box binding protein

**TF**, transcription factor

**TTD**, trichothiodystrophy

**VRK1**, Vaccinia‐related kinase 1

**WIP**, wild‐type phosphatase

**XPC**, Xeroderma pigmentosum complementation group C

**XRCC1**, X ray repair cross‐complementing protein 1

DNA, the basic unit of genetic information, is approximately two metres long and is tightly packed around positively charged histone proteins. Histones bind electrostatically to the negatively charged phosphate groups of DNA, forming nucleosomes, the basic unit of chromatin. Each nucleosome consists of an octameric core of histones (H2A, H2B, H3 and H4), while linker histone H1 (in eukaryotes) and H5 (a variant of H1, found in avian and reptilian species) stabilize higher‐order chromatin conformation [1]. This compact structural state of chromatin restricts access to DNA and thus modulates various biological processes, including transcription, replication and DNA repair [2, 3].

Chromatin accessibility is dynamically regulated by post‐translational modifications (PTMs) on histones, which either serve as docking platforms for chromatin remodellers and histone chaperones or alter their electrostatic properties [4, 5]. These modifications, which include PARylation, phosphorylation, acetylation, methylation, SUMOylation, crotonylation, succinylation, isomerization and citrullination, play a pivotal role in DNA‐related functions. One of their major roles is regulating transcription in response to DNA damage, ensuring transcription recovery and genome stability.

### Transcription regulation in eukaryotes

In eukaryotes, transcription initiation begins with TFIID binding to the TATA sequence of the DNA via the TBP (TATA‐binding protein) subunit, followed by the sequential recruitment of general transcription factors (TFIIA, TFIIB, TFIIE, TFIIF and TFIIH) and RNA polymerase (Pol II) [6], finally forming the pre‐initiation complex (PIC) to initiate the synthesis of RNA using DNA as a template [7]. Following initiation, Spt5/4 (DSIF, DRB sensitivity‐inducing factor) and negative elongation‐inducing factor (NELF) transiently pause RNA Pol II. Transcription elongation is then triggered by the phosphorylation of RNA Pol II C‐terminal domain (CTD) at serine 2 by the catalytic cyclin‐dependent kinase 9 (CDK9), a subunit of the positive transcription elongation factor (P‐TEFb) [8, 9].

The tight association of histone tails with DNA presents a physical barrier to the progression of RNA Pol II. However, histone PTMs and chromatin remodellers, such as facilitates chromatin transcription (FACT) complex, can loosen the contact between DNA and histone, therefore facilitating RNA Pol II passage and later reassembly of the nucleosome [10, 11, 12].

### Transcription regulation in the DNA damage response (DDR)

Transcription inhibition has been recognized as an indicator of DNA damage because DNA lesions physically obstruct ongoing transcription. Conversely, the recovery of transcription has been considered a surrogate marker of DNA repair. However, the relationship between DNA damage, DNA repair and transcription is much more complex due to the various direct and indirect effects of DNA damage on transcription.

The transcriptional outcomes following DNA damage depend on the type and location of the DNA lesion. Non‐bulky lesions such as the oxidative base lesion 8‐oxoguanine (8‐oxoG), DNA nicks, or single‐stranded gaps can be bypassed by the RNA Pol II machinery, leading to transcriptional mutagenesis and affecting cellular function. When such mutations occur within a gene regulatory sequence, they could prevent the binding of transcription factors, resulting in the misregulation of the target genes [13]. In contrast, helix‐distorting bulky DNA lesions or cyclobutene pyrimidine dimers (CPDs) occurring in the transcribed region of genes, caused by UV radiation or cross‐linking agents, can stall RNA Pol II progression, triggering a cascade of nucleotide excision repair (NER) and transcriptional recovery events. Defects in factors responsible for the removal of transcription‐blocking lesions (TBLs) can lead to various disorders, such as Cockayne syndrome (CS), trichothiodystrophy (TTD), neurodegenerative diseases, infectious diseases and inflammation, highlighting the importance of understanding DNA damage‐induced transcription regulation [14, 15, 16, 17, 18]. The Cockayne syndrome B (CSB) protein, which is a DNA‐dependent ATPase, promotes RNA Pol II progression under normal conditions but fails to resolve TBLs, leading to transcriptional suppression [19]. Subsequent recruitment of the CSA‐DDB1‐CRL4 ubiquitin ligase complex facilitates RNA Pol II ubiquitylation on K1268 of the RPB1 subunit, serving as a marker for repair [20, 21]. The DNA repair factor ultraviolet‐stimulated scaffold protein A (UVSSA) then mediates RNA Pol II backtracking, enabling the exposure of DNA lesions and recruits the NER machinery, including TFIIH [22, 23]. TFIIH not only facilitates DNA repair but also plays a prominent role in transcriptional recovery through CDK7‐mediated phosphorylation on serine 5 of RNA Pol II [24]. Additionally, the FACT complex assembles the nucleosome for the RNA Pol II to move forward, thus aiding in transcription elongation recovery [25, 26].

Another major cause of transcription stalling is DNA–protein crosslinks (DPCs). These covalent adducts act as a barrier to the progression of RNA polymerase II transcription and stall DNA replication. Stalling of RNA Pol II after DPC formation initiates transcription‐coupled (TC) DPC repair by recruiting CSB and CSA proteins [27, 28, 29, 30]. To ensure proper replication after DPC in mammals, SPRTN, a replication‐specific protease, digests the proteinaceous part of the DPC, protecting from the replication fork collapse [31]. DNA damage can also occur during the process of transcription itself. Nascent RNA binds to the template DNA strand forming a short DNA–RNA hybrid or a three‐stranded structure, known as an R‐loop. These structures accumulate during transcription initiation and termination, and block the progression of RNA Pol II, causing DNA damage and threatening genome stability [30]. The TC‐NER mechanism, and other enzymes such as RNase H, helicases and topoisomerases are involved in resolving these R‐loop structures and maintaining genome integrity [32].

DNA damage can regulate transcription both *in cis* and *in trans*. Many DDR genes, including the tumour suppressor protein p53, are activated in response to the blockage of transcription. Following its activation, p53 accumulates in the nucleus and functions as a transcription factor to induce the transcription of downstream genes. Depending on the cell type, damage type and severity, p53 can enhance cell survival by upregulating DNA repair genes and cell cycle inhibitors or induce cell death by activating apoptotic genes. In both scenarios, the mutagenesis and cytotoxicity caused by DNA damage can be mitigated [33].

Due to the role of histone modifications in regulating gene expression, DNA repair and chromatin architecture, their dysregulation can contribute to various disorders such as cancer, neurodegenerative diseases, ageing and other metabolic disorders [34, 35, 36]. Several examples, such as uncontrolled H3 and H4 acetylation, can influence transcription silencing, contributing to Huntington's disease. Loss of H4K16ac contributes to defective DNA repair, resulting in Werner's syndrome and progeria. Impaired γ‐H2AX at the site of DNA damage due to mutated ATM can cause ataxia‐telangiectasia. Abnormal histone acetylation levels are observed in peripheral blood mononuclear cells of Type 2 diabetes patients, suggesting this modification's involvement in the pathogenesis of metabolic diseases. These studies suggest the role of histone modifications in DNA damage‐related pathogenesis.

**Fig. 1:**
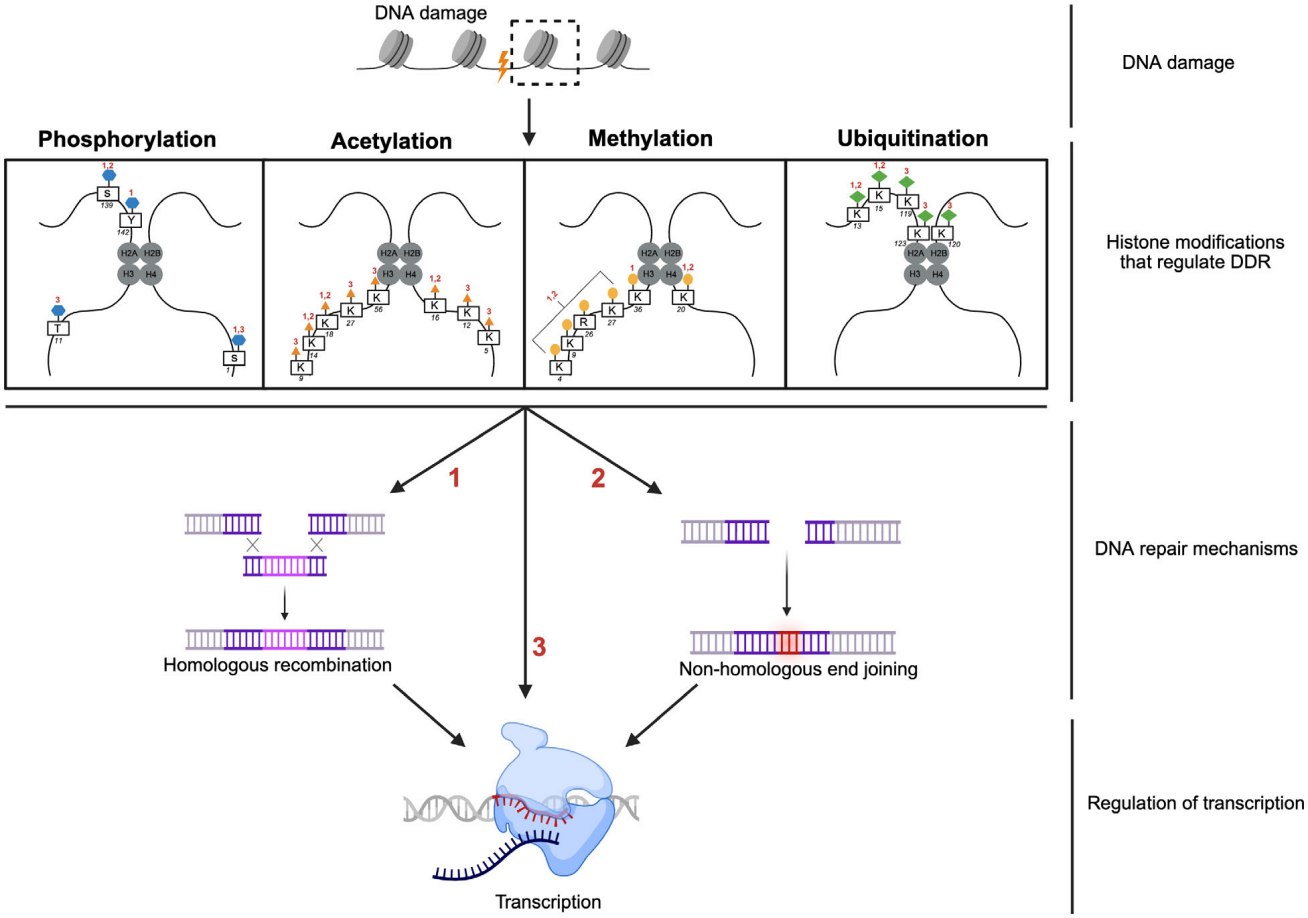
Schematic representation of various types of histone modifications in transcription regulation during the DDR. Created in BioRender. Wang, S. (2026) https://BioRender.com/4gf9os7.

This review explores the role of histone modifications in regulating transcription during the DDR Fig. 1, with a focus on their mechanistic contributions to damage recognition, repair and transcriptional recovery. We also discuss the implications of dysregulated histone PTMs in ageing‐related diseases and highlight emerging modifications in DDR that warrant further investigation.

## Histone PARylation

Histone poly(ADP‐ribosyl)ation or histone PARylation is a post‐translational modification where ADP ribose polymers are attached to histone proteins. This modification is primarily catalysed by Poly ADP Ribose polymerases (PARP). The catalytic domain (CAT) of PARP proteins binds nicotinamide adenine dinucleotide (NAD+) catalysing the PARylation of target proteins, including histones [37]. The PARP family of proteins consists of 17 members, out of which PARP1 and PARP2 are the most abundant forms involved in DNA damage, replication, transcription and apoptosis [38]. PARP‐1 contains 6 domains including two homologous zinc finger domains (Zn1 and Zn2) followed by a Zn3 and a WGR (Trp‐Gly‐Arg) domain [38, 39]. Between the Zn3 and the WGR domains, there is the auto‐modification domain (AD) which contains the BRCA1 C terminus (BRCT) fold that recognizes intact DNA and mediates the interaction with DNA repair proteins [40]. Functionally, it facilitates DNA repair by stimulating base excision repair (BER), non‐homologous end joining (NHEJ) and homologous recombination (HR) [41].

### Histone PARylation in the DDR

During DNA damage, PARP's Zn1 and Zn2 domains recognize the damage site by forming either a ‘base stacking loop’ with the exposed nucleotides or a ‘backbone grip’ with the DNA phosphate backbone [41]. This interaction causes a conformational change in PARP's CAT domain, making NAD+ accessible and forming negatively charged PAR chains at DNA damage sites [33]. PAR chains can open the chromatin and enable DNA repair, by recruiting proteins such as X‐ray repair cross‐complementing protein 1 (XRCC1) [42], xeroderma pigmentosum C (XPC) [43] and meiotic recombination 11 (MRE11) or Nijimegan breakage syndrome 1 (NBS1) [44] in single‐stranded breaks (SSBs), UV‐induced photolesions and double‐strand breaks (DSBs), respectively. In the presence of DSBs, PARP1 plays a crucial role in regulating DNA repair pathway choice. At the DSB site, PARP1 competes with the Ku70/80 complex during the S/G2 phases, promoting alternative NHEJ, or interacts with DNA‐PKcs, resulting in classical NHEJ [45]. Reports also show that MRE11 interacts non‐covalently with PAR chains, which is essential for HR [45].

Excessive PARP activity leads to NAD + depletion due to its over consumption, leading to neurological and mitochondrial dysfunction, as well as ageing‐associated diseases [46, 47, 48]. Thus, it is essential to suppress PARP activity after DNA lesion recognition. Recent reports suggest that the functions of PARP1 and XRCC1 are reciprocal upon genotoxic stress. To prevent overactivation of PARP activity and restore chromatin stability after DNA repair, the recruitment of XRCC1 dissociates PARP from the DNA [49]. Lee *et al*. [50] show that the Ewing sarcoma protein (EWS) is required for the proper dissociation of PARylated PARP1 from DNA damage sites after the recruitment of the DNA repair proteins. Thus, auto‐PARylation and PARylation of other proteins is a highly regulated process involved in the maintenance of genomic stability.

### PARylation in transcriptional regulation during the DDR

The PARP protein family plays a vital role in regulating transcription and maintaining genome integrity, by chromatin remodelling [51, 52], interacting with transcription factors, displacing the RNA polymerase machinery during damage and through crosstalk with other post‐translational modifications [53].

Immediately after DNA damage, it is important to pause ongoing transcription to prevent collisions between the transcriptional and DNA repair machineries [54]. PARP1 auto‐PARylation produces PAR structures that recruit PcG and NuRD complexes to the sites of DNA damage [55, 56]. These transcriptional repressor complexes evict RNA Pol II from the chromatin to prevent inefficient transcription and the release of premature transcripts [55, 57]. Another report suggests that PARP1 binds to P‐TEFb upon DNA damage, preventing cyclin—T1 from undergoing liquid–liquid phase separation thereby stopping CDK9 from phosphorylating RNA Pol II serine 2 and resulting in transcription elongation inhibition [57]. Therefore, PARP1 acts as a quality control factor to prevent the release of immature transcripts.

On the contrary, PARP1 regulates remodelling of the chromatin and recruitment of DNA repair factors at the damaged sites to facilitate transcription *in cis*. Immediately upon DNA damage, PARP1 is activated and it PARylates the KDM5B demethylase for H3K4me3, excluding KDM5B and opening the chromatin to allow DNA repair [58]. Meanwhile, PARylation of PARP1 can also dissociate H1 from the promoter regions of the genes. Dissociation of H1, a repressor of RNA polymerase II‐mediated transcription, facilitates active chromatin for DNA repair and thereby active transcription [59]. At the time of DNA damage, the KDM4D demethylase is PARylated on its C‐terminal domain, to the site of DNA damage, thereby increasing the demethylation activity of H3K9, facilitating open chromatin and promoting DSB repair [60, 61, 62] in the neurons of the hippocampus and amygdala.

Beyond the role in chromatin remodelling, PARP interacts with various transcription factors to modulate the transcriptional activation of DDR genes. Post DNA damage, the NF‐κB p65‐p50 heterodimer is generally translocated to the nucleus [63, 64]. PARP1‐mediated PARylation at the damage site causes the release of the negatively charged PAR chains that directly bind p65, stabilizing the interaction between NF‐κB and the transcription machinery [18]. This facilitates the pre‐initiation complex formation and transcription activation, thereby enhancing transcription initiation of NF‐κB‐dependent anti‐apoptotic genes [64]. PARP1 also acts as a transcription co‐activator via interacting with or PARylating various transcription factors. For instance, it physically binds the promoter of the transcription factor E2F and upregulates its expression [65]. E2F, in turn acts as a transcription activator after being phosphorylated by ATM or ATK, recruits XPA to the site of damage and facilitates the retention of other repair factors [66, 67]. PARylation generally produces a dual response either by regulating transcription factors or cell cycle checkpoints [68]. AutoPARylated PARP1 binds to p53, promoting covalent PARylation and recruiting p53 to the DNA damage site. This paves the way for the recruitment of 53BP1 and DDB1 to the damage and facilitates HR and NHEJ, highlighting the role of PARylation in activating DNA repair and transcription [69]. PARP1 activation upon DNA damage can activate the PLK3 gene in an ATM‐mediated manner and phosphorylate p53, affecting its transcriptional activity [70], essential for p53‐dependent apoptosis [71].

PARylation engages in crosstalk with other histone PTMs to orchestrate the transcriptional response to DNA damage. γ‐H2AX, known as the primary histone marker of DSBs, recruits PARP1 to the damaged site. This association enhances the catalytic activity of PARP1 and initiates the first step of DNA repair [72]. A recent study shows that histone deacetylase 5 (HDAC5)‐mediated histone deacetylation modulates PARP1's DDR and facilitates the efficient recruitment of DNA repair factors following damage [73].

Overactivation of PARP1 causes various ageing‐related disorders, such as osteosclerosis, neurodegeneration, Parkinson's disease and Alzheimer's disease [74, 75]. To prevent excessive activation of PARP1 after DNA repair is completed, RNF146 uses its WWE domain to recognize and bind PAR chains, which leads to the ubiquitination and subsequent degradation of PARP1 [76]. Inhibition of PARP1 can lead to the accumulation of RNA Pol II at the post‐damage sites, indicating the importance of PARylation for efficient transcription recovery [77].

The role of histone PARylation in DDR is highly exploited in cancer therapeutics. FDA‐approved PARP inhibitors such as Olaparib, Rucaparib, Talazoparib and Niraparib are used to block PARP1 activity, thus accumulating DNA damage and causing cell death in breast cancer type 1 susceptibility protein (BRCA 1)‐deficient cancer cells that have a defective HR [78]. PARP inhibitors are also used in combination with chemotherapy, radiotherapy and immunotherapy to enhance the effectiveness of the treatment and overcome the development of resistance to treatment by the tumour cells. Due to the various functions of PARP1 and its associated proteins, it will be interesting to explore how PARP balances its dual role as a transcription activator and repressor upon DNA damage.

## Histone phosphorylation

Histone phosphorylation is a process by which protein kinases, such as PKA, CDK and ATR, add negatively charged phosphate groups from ATP to the hydroxyl group of amino acid residues such as serine, threonine, tyrosine on the N terminus of histone tails [5, 79] thereby promoting transcription activation [80]. Histone phosphorylation can alter chromatin structure, serving as a platform to recruit various DNA repair proteins and transcription factors, ensuring genome stability [79].

### Histone phosphorylation in the DDR

Among all the histone PTMs, H2AX phosphorylation (*γ*‐H2AX) serves as the most established biomarker of DNA damage. H2AX phosphorylation at serine 139 is a rapid response that occurs within seconds following DSB generation [81]. Initial damage recognition involves NBS1 recruitment to the damage site through its interaction with the MRN complex (MRE11‐RAD50‐NBS1) [82]. Subsequently, ATM phosphorylates NBS1 and H2AX at their N terminus, while activating downstream transcription and cell cycle check point regulators. *γ*‐H2AX stabilizes the MRN complex and recruits repair proteins such as 53BP1 and RAD51 to the damage site, thereby initiating NHEJ and HR repair pathways [83, 84]. Notably, during infrared irradiation, Vaccinia‐related kinase 1 (VRK1), a chromatin kinase in cooperation with ATM, phosphorylates H2AX at serine 139 [85]. *γ*‐H2AX‐deficient mice exhibit growth retardation, chromosome instability and DNA repair deficiency, underscoring the crucial role of *γ*‐H2AX in genomic maintenance [86].

After DNA repair completion, it is vital to remove the phosphorylation of H2AX to prevent cellular senescence and ageing [87]. Dephosphorylation of *γ*‐H2AX by wild‐type p53‐induced phosphatase 1 (WIP1) prevents further recruitment of DNA repair proteins such as 53BP1 to the lesion site, ensuring the completion of DNA repair [88]. Another approach to remove *γ*‐H2AX from the damage site happens through the recruitment of BRCA1. BRCA1 interacts with H2AX to form a stable biochemical complex. This complex facilitates ubiquitination of H2AX at lysine 119, thus attenuating *γ*‐H2AX level [89]. It is hypothesized that the interaction between BRCA1 and *γ*‐H2AX ensures timely suppression of the active repair phase. Studies indicate that H4S1 phosphorylation mediated by casein kinase II occurs within 1 kb of the DSB site, after NuA4 activity, which is involved in recruiting DNA repair proteins during NER. Thus, H4S1 phosphorylation after the action of NuA4 and its correlation with chromatin deacetylation, which blocks further action by NuA4, suggests its role in chromatin restoration after DNA repair [90].

### Histone phosphorylation in transcriptional regulation during the DDR

Histone phosphorylation is critical for regulating transcription in response to DNA damage, resulting either in transcription attenuation at the damage sites or chromatin remodelling to activate DDR‐related transcription [91]. In addition, it regulates transcription via phosphorylating other histone modifying enzymes in an ATM/ATR‐dependent manner [92].

As described earlier, immediately after DNA damage, *γ*‐H2AX is recruited to the DSB site and retained due to the autophosphorylation of ATM kinase at serine 1981. Although ATM autophosphorylation is not required for *γ*‐H2AX, ATM kinase activity itself is still essential [93]. Following *γ*‐H2AX, mediator of DNA damage checkpoint 1(MDC1) is recruited and binds to *γ*‐H2AX via its BRCT domain, thereby stabilizing the damage foci [94]. MDC1 in turn recruits RNF8‐E3 ubiquitin ligase, which mono‐ubiquitinates H2A and H2AX, facilitating RNF168 binding and enhancing ubiquitination [79]. This amplified *γ*‐H2AX‐mediated ubiquitination signal serves as a platform for transcription repressor complexes to bind and silence transcription at the damage site [95]. Evidence suggests that the absence of H2A ubiquitylation, caused by mutated RNF8, can rescue transcription [95].

The dissociated 53BP1 from the Tudor‐interacting repair regulator (TIRR)/53BP1 complex [96] binds to the ubiquitinated H2AX foci at the damaged site, leading to the RNA Pol II dissociation. Mechanistically, 53BP1 engages in crosstalk with H4K20me2 via its Tudor domain [97], leading to chromatin compaction and preventing the RNA Pol II machinery from moving forward, thereby causing its dissociation from the chromatin [98, 99, 100]. In addition, 53BP1 can further enhance chromatin compaction and RNA Pol II dissociation by stabilizing histone deacetylase 1 (HDAC1) [98]. These pathways play a key role in regulating transcription by compacting chromatin in a timely manner and arresting transcription elongation at damaged sites. After the completion of DNA repair, *γ*‐H2AX is dephosphorylated by PP2A phosphatases, preventing transcriptional silencing and thus promoting transcriptional recovery [101].

Beyond the *γ*‐H2AX‐mediated local transcriptional silencing at damaged sites, other histone phosphorylation events can activate the transcription of certain DDR genes. For instance, ERK kinase‐mediated H3S10 phosphorylation induces chromatin decompaction and activates the transcription of DDR genes immediately following genotoxic stress [102]. It was shown that H3S10 phosphorylation negatively correlates with H3K9me2, a mark for transcriptional repression, while co‐occurring with active transcription marks including H3K4me3, H3K9ac and H3K27ac. Thus, H3S10 phosphorylation associates with enhanced transcription elongation [103]. Additionally, H3S28 phosphorylation activates the transcription factors such as C‐FOS and JUN following UV‐induced DNA damage or other stressors [104].

Aberrant histone phosphorylation is associated with genome instability and several disorders [105]. Studies show that elevated levels of *γ*‐H2AX are associated with neurodegenerative diseases [106]. *γ*‐H2AX is also a widely used biomarker of DNA damage in cancer diagnosis and treatment. A significant increase in *γ*‐H2AX expression is found in gastric carcinoma [107], lung cancer [108], melanoma [109] and colon cancer [110]. In colorectal cancer, *γ*‐H2AX expression highly correlates with the tumour's malignant behaviour and poor survival of the patients [111]. Importantly, γ‐H2AX is not always ‘bad’; in fact, it represents a necessary and beneficial step in the DDR of healthy cells. However, the role of γ‐H2AX is highly context‐dependent. In pre‐cancer cells, if DNA damage persists and γ‐H2AX foci remain unresolved, this persistence can promote carcinogenesis by promoting genomic instability. Once a tumour is established, γ‐H2AX accumulation can act in two opposing ways. On one hand, it can be advantageous, since sustained signalling may cause replication arrest or trigger cell death, thereby suppressing tumour growth. On the other hand, it can also be detrimental, as chronic γ‐H2AX signalling may contribute to further mutations and genomic rearrangements that enhance the metastatic potential of cancer cells. Precancerous lesions have high levels of DSBs due to active oncogenes stalling DNA replication forks [112]. Thus, due to the levels of DSBs, *γ*‐H2AX is used as a biomarker to detect these lesions and take preventive measures. Moreover, telomere shortening is also correlated with *γ*‐H2AX foci formation in ageing and cancer cells. Studies have found that triple‐negative breast cancer (TNBC) has shortened telomeres, and *γ*‐H2AX is exploited as a prognostic biomarker for TNBC [113]. Apart from *γ*‐H2AX, defective H3S10 and H3S28 phosphorylation also associate with dysregulation of transcription, contributing to carcinogenesis [103, 114].

In the context of cancer therapy, *γ*‐H2AX can be used to indicate the efficiency of the treatment. Preoperative radiotherapy, a standard therapy for rectal cancer, prevents the local recurrence of the disease. *γ*‐H2AX is used to correlate with the radiosensitivity of patients with colorectal cancer [115]. Another study indicates its role as the first molecular marker identified that can reveal the survival heterogeneity in *γ*‐H2AX‐positive breast cancer [116]. Thus, elevated *γ*‐H2AX can be used both as a marker of cancer and an indicator of cancer therapy, and targeting *γ*‐H2AX has the potential to be used in combination with other therapies to increase therapeutic efficiency.

## Histone acetylation

Histone acetylation is a process by which acetyl groups are added to the lysine residues of histone tails. This post‐translational modification can neutralize the positive charge on the lysine residue, thereby weakening the interactions between histones and DNA and contributing to opening the chromatin, and making it transcriptionally active [117]. Acetylation, which is catalysed by histone acetyl transferases (HAT), increases chromatin accessibility for the RNA polymerase II machinery, DNA repair proteins and other transcription factors [118]. On the contrary, histone deacetylases (HDACs) are involved in the deacetylation of histones post‐repair, restoring the positive charge to the histone and making the chromatin more compact to stabilize the genome.

### Histone acetylation in the DDR

Histone acetylation regulates the DDR by detecting the damage, condensing the chromatin to repress transcription, opening chromatin to recruit DNA repair machinery and restoring transcription.

Upon DNA damage, histone acetylation plays a role in chromatin remodelling for efficient DNA repair [119]. Once *γ*‐H2AX occurs at the damage site, H4 acetylation causes dynamic changes in the chromatin by disrupting the inter‐nucleosomal interactions, resulting in the opening of chromatin, recruiting an optimum level of 53BP1 and enhancing DNA repair [117]. H4 and H2A acetylation mediated by the NuA4‐TIP60 complex is vital to transform the chromatin from its repressive state to an active one, providing a platform for DNA repair. After DNA damage is sensed, NuA4 is recruited through a direct interaction with the MRX complex and it spreads along the DNA resection ends. It has an antagonistic regulatory function on NHEJ factors, favouring an alternative choice of DNA repair pathway [120]. It is shown that NuA4‐TIP60 mutants can cause defective repair and increase genome instability. In addition, the TIP60 complex is involved in recruiting H2AZ, a variant of H2A, modulating gene expression and altering the chromatin structure locally favouring DNA repair [121]. H4K16 acetylation (H4K16ac) by MOF exhibits a biphasic pattern, undergoing deacetylation immediately after damage, followed by acetylation to modulate 53BP1 recruitment. These biphasic dynamics suggest that H4K16ac plays an important role in fine‐tuning the level of 53BP1 after DNA damage [122]. Thus, H4K16ac is a key regulator that controls the choice of the repair pathways through regulating 53BP1 recruitment. Elevated H4K16ac activates ATM and promotes resection that facilitates HR, while lower levels can attenuate resection, proceeding towards NHEJ [123]. Meanwhile, other histone acetylation modifications such as H3K18ac [124, 125] and H2AK15ac [126], favour and promote NHEJ and HR respectively. The immediate *γ*‐H2AX also orchestrates with other histone acetylation marks. A negative correlation between *γ*‐H2AX and the level of H3K9ac and H3K56ac was observed [127]. Since *γ*‐H2AX upon damage recruits a HAT enzyme, KAT2A, it can be hypothesized that *γ*‐H2AX could indirectly influence the restoration of H3K9ac and H3K56ac marks. This suggests that *γ*‐H2AX ensures transcription repression at the damaged sites, followed by chromatin opening mediated by H4K16ac, the recruitment of DNA repair proteins, and H3K56 hypoacetylation to promote NHEJ [128].

Once the chromatin opens, H3K14ac allows the retention of the chromatin structure remodelling complex (RSC) and enhances the removal of cyclobutane pyrimidine dimer (CPD) by DNA repair proteins [129]. Evidence suggests that histone chaperone anti‐silencing factor 1(ASF1) can lead to a crosstalk between H3K14ac and H3K56ac [130]. ASF1‐bound H3 can better serve as a substrate for GCN5‐mediated H3K14ac and further acetylate H3K56 [130]. Atomistic molecular dynamics simulations show how H3K56ac exposes DNA damage sites for lesion sensing, making it accessible to DNA repair proteins [131]. Loss of H3K14ac exhibits a decrease in the level of H3K56ac, causing genome instability [130].

After the repair process is completed, restoring the chromatin is essential to maintain its compact and condensed state, which can otherwise lead to cancer, neurodegeneration and ageing [132]. HDAC1 and HDAC‐2 are recruited to the DSB sites after repair is completed, where they remove the H3 and H4 acetylation marks [133]. H4K16ac is removed to the baseline to maintain the chromatin structure. In yeast and mammals, H3K56ac is removed to facilitate assembling new nucleosomes after repair, stabilizing the chromatin [134, 135].

### Histone acetylation in transcriptional regulation during the DDR

While histone acetylation‐mediated chromatin relaxation is essential for allowing DNA repair proteins' access to lesion sites, the resulting open chromatin state can create a conflict between transcription and repair machineries. This interference may compromise repair fidelity or generate aberrant transcripts. Therefore, beyond its chromatin remodelling function, histone acetylation also plays a critical role in establishing and maintaining transcriptional repression during the DDR.

As the first step of the DDR, loss of certain histone acetylation marks on the DNA damage site triggers transcription repression. [136]. H3K9ac marks, under untreated conditions, provide active transcription. Upon DNA damage, ATM activation phosphorylates H2AX, MDC1 and KAP1 (a transcriptional co‐repressor) [137]. Phosphorylation of KAP1 on serine824 recruits the NuRD complex consisting of HDAC1and HDAC2 and causes deacetylation of H3K9 and transcription silencing at the damage site [137, 138, 139]. Another study indicates that loss of H3K27ac, an active chromatin mark, immediately after micro‐irradiation at the DSB site, contributes to silencing transcription [140]. To facilitate faithful DNA repair, it is essential to prevent the collision of DNA repair and premature transcripts. Hence, it is essential to regulate premature transcription termination near DNA lesions. In yeast, H3K4me3 promotes recruitment of Nrd1, responsible for efficient termination in a Set1‐dependent manner [141]. While loss of Set1 correlates with increased acetylation and termination defects, the role of histone acetylation in efficient regulation of transcription termination can be speculated.

Furthermore, to facilitate DNA repair, histone acetylation marks can regulate transcription initiation, elongation and termination processes of DNA repair genes. In response to p53 activation, H3 and H4 acetylation on the promoters reshapes the chromatin and releases RNA Pol II from the promoter, promoting the transition from transcription initiation to elongation [142, 143]. H4K16ac deposition by MOF elevates the expression of repair proteins, through recruiting readers such as bromodomain‐containing protein 4 (BRD4). BRD4 can recruit the p‐TEFb complex, which phosphorylates RNA Pol II CTD at serine 2 and leads to the release of paused Pol II, allowing it to proceed to elongation [144]. BRD4 is also a master regulator of transcriptional recovery through re‐engaging RNA Pol II. This suggests that H4K16ac can regulate transcription activation of repair genes and restore recovery of transcription post‐repair. Post‐repair, H3K9ac and H3K27ac are restored and recruit the TFIID subunit of the RNA Pol II pre‐initiation complex to the gene promoters, thereby restoring the initiation of transcription [145].

In the context of diseases, H3K27 hyperacetylation decreases the activity of HDACs, sirtuins, as well as causing transcriptional dysregulation during Parkinson's disease pathogenesis [146]. Defects in various subfamilies of HATs like CREBBP, EP300, KAT6A, KAT6B and histone deacetylase HDAC4 cause several disorders such as Rubinstein–Taybi Type 1 and Type 2 syndromes, KAT6A syndrome, genitopatellar syndrome, Say–Barber–Biesecker–Young–Simpson syndrome and brachydactyly mental retardation syndrome [147], respectively. They are mostly congenital diseases that are caused due to dysregulated transcription. In the context of DNA damage‐related diseases, dysregulated histone acetylation patterns, primarily contribute to cancer, by leading to aberrant expression of genes involved in the cell cycle, apoptosis and tumour differentiation. Studies indicate that an increase in H3K4ac in promoter regions of EMT marker genes, involved in the hedgehog signalling pathway, facilitates cancer cell migration and invasion in head and neck cancer [148]. Another study shows a negative correlation of SIRT1 and H3K4ac in breast cancer cell lines. Thus, HDAC1 can be targeted to affect SIRT1 function as a therapy for breast cancer [149]. Sirtuins, a well‐known protein family pertaining to class III HDACs, is utilized as a therapeutic in ageing and longevity. Reports suggest that caloric restriction activates sirtuins, reducing histone acetylation and increasing lifespan in mice, deacetylating H4K16ac at pro‐apoptotic genes and reducing inflammation as well as acting as a tumour suppressor [150, 151]. Hence, this indicates the possibility of regulating histone acetylation markers towards therapeutic approaches.

## Histone methylation

Histone methylation is the addition of one, two, or three methyl groups to lysine or arginine residues in histone tails [152]. Methylation occurs in both heterochromatic and euchromatic regions and is involved in transcription activation, transcription repression and DNA repair [153]. Methyl groups are added by specific histone lysine methyltransferases (KMTs), with S‐adenosylmethionine (SAM) acting as a methyl group donor [154], and removed by histone demethylases (KDMs). Although histone methylation cannot directly alter the electrostatic property of chromatin, it can influence how proteins interact with it through recruiting various readers that recognize this modification; therefore, it plays a crucial role throughout the DDR process.

### Histone methylation in the DDR

Unlike histone acetylation, which is generally involved in the opening of chromatin and transcriptional activation, histone methylation plays a dual role in transcription regulation. Its effects depend on both the specific modified residue and the methylation states, enabling either active or repressive chromatin states. Due to its functional variability, histone methylation is involved in multiple DDR processes, including recognizing DNA damage, opening chromatin, recruiting DNA repair proteins and restoring post‐repair transcription. Dysregulation of histone methylation leads to genome instability, cancer and other age‐related disorders.

H3K4me3 is an active transcription mark. Immediately following DNA damage, demethylation of H3K4me3 by KDM5B is essential for repressing local transcription and compacting chromatin by recruiting the zinc finger MYND‐type containing 8 (ZMYND8)‐NuRD chromatin remodelling complex. H3K4me3 on *γ*‐H2AX sites can impair the recruitment of DNA repair proteins [155], and loss of KDM5B prevents activation of both NHEJ and HR repair pathways [156]. Thus, H3K4me3 is one of the first events post DNA damage, in yeast and mammals, accumulating at the DSB sites [144]. Later on, H3K4me3 is increased on specific cell cycle genes to induce cell cycle arrest [157]. In addition, a study in *C. elegans* shows that H3K4me2 deposition is increased upon the completion of DNA repair and regulates the recovery of transcription and protein biosynthesis (a topic that will be further discussed in the next section) [158]. In mammals, loss of LSD1, a demethylase enzyme for H3K4me1/2, can promote HR in response to DSBs [159, 160]. These studies show how different states of methylation on the same amino acid residue exhibit different functions during the DDR. Thus, further investigation on how H3K4 methylation and the HMT, MLL‐COMPASS complex are regulated in DDR is required.

Apart from H3K4 methylation, other histone methylations exert different functions during the DDR. H3K9 methylation protects genome integrity by maintaining the heterochromatin organization [161] without compromising DNA repair efficiency. Heterochromatin presents a physical barrier to the DNA repair machinery; thus, demethylation of H3K9 is a crucial step for ensuring efficient DNA repair. Loss of KDM4B, which is responsible for demethylating H3K9me3 and H3K9me2, can increase persistent *γ*‐H2AX, suggesting KDM4B acts as a DDR protein to enhance repair and confer resistance after radiation [62]. One of the most understudied histone methylation marks is H3K79me, which occurs in the globular domain of H3, rather than its histone tail. Upon damage, 53BP1 recognizes DOT1L‐mediated H3K79me2 via its Tudor domain and then blocks resection to promote NHEJ [162, 163]. It seems that H3K79me2 is involved in choosing the repair pathway; however, whether removal of H3K79me2 promotes the engagement of an error‐free repair pathway is not yet studied. Also, H4K20 methylation plays a fundamental role in the DNA repair process. SETD8 is required to monomethylate H4K20, which is further methylated by SUV4‐20H1 and SUV4‐20H2 leading to di‐ or trimethylation. After UV irradiation, SET8 is degraded by CRL4 ubiquitin ligase in a PCNA‐dependent manner, resulting in the degradation of H4K20me1 to control cell proliferation [164]. An alternate pathway to regulate DNA repair by H4K20 methylation is by recruiting 53BP1. Upon damage, the tandem Tudor domain of 53BP1 recognizes H4K20me2 favouring NHEJ. H2AK15ub is important to recruit 53BP1 upon damage, and thus, the dual interaction with these histone marks helps in the retention of 53BP1 at the damage foci, contributing to cell cycle arrest at the G2/M phase [165]. The crosstalk between H3K79 methylation and H4K20 methylation to recruit 53BP1 and whether loss of H4K20 methylation exhibits a bias towards HR have not been investigated.

### Histone methylation regulating transcription during the DDR

Histone methylation serves as a dual‐function chromatin mark that either represses transcription upon damage or promotes initiation and elongation of transcription during and post DNA repair. Upon DNA damage, TIP60 acetyltransferase, mediated by ATM signalling, allows access of SUV39H1 to deposit H3K9me2 and H3K9me3 at the DSB sites. These marks can recruit HP1 and cause chromatin compaction, making the RNA Pol II machinery dissociate from the promoters, leading to transcription repression at the break [166]. Another way to silence transcription locally at the damage site is mediated by polycomb repressive complex (PRC). Enhancer of zeste homologue (EZH2), a subunit of PRC2, can deposit H3K27me3 on the transcribing regions of the damage sites and silence the ongoing transcription, providing time for DNA repair [167]. However, whether H3K9me2/3 and H3K27me3 complement each other to repress transcription is not yet studied.

During the next phase of DDR, which is DNA repair, the transient deposition of the transcription silencing marks is removed, to maintain the open chromatin structure and facilitate DNA repair. KAP1, upon damage acts as a scaffold protein, and interacts with HP1 and CHD3. ATM activation upon DNA damage phosphorylates KAP1 at serine 824, which in turn opens the heterochromatin architecture and temporarily loosens the chromatin for repair [168]. Loss of KDM4A demethylase for H3K9me3 is proved to show a delay in transcription restoration post‐repair. Simultaneously, KDM6A erases H3K27me3, enabling the binding of TFIID to the RNA Pol II and the promoters, and facilitates transcription recovery post‐repair [95, 169]. Loss of both repressive marks can prevent transcriptional silencing after the completion of repair.

Along with the removal of the repressive marks, it is essential to re‐establish the active histone methylation marks, to regulate transcriptional recovery. H3K9 demethylation provides a transcription‐friendly chromatin environment, facilitating MLL‐COMPASS to deposit H3K4me3 and restore transcription. In yeast, it has been shown that H3K4me3 is recruited at sites of DSBs and facilitates repair, allowing for transcriptional recovery [144]. Removal of H3K9 methylation upregulates the expression of DNA repair factors such as BRCA1, and RAD51 for HR while it could also be one of the required factors for the methylation of H3K4 [170]. A further mechanism to elucidate the crosstalk between the repressive H3K9 methylation and the active H3K4 methylation in the context of post‐repair transcription recovery can be investigated. In addition, in *C. elegans*, deposition of H3K4me2 is observed at 24 h post DNA damage at the gene bodies [158], and it is correlated with transcriptional recovery without affecting the DNA repair process. This creates the intriguing question of whether H3K4me2 is a general marker for transcriptional recovery in response to transcription stress without DNA damage.

H3K36me3 is associated with transcription elongation and is generally deposited on the 3′ ends of genes. Chromatin opening mediated by ATM/ATR signalling re‐engages SET1D to deposit H3K36me3 [171, 172]. This mark along the gene bodies, further recruits SPT16, causing serine 2 phosphorylation at the CTD of RNA Pol II and stabilizing the elongation complex, hence resuming transcription elongation specifically post‐repair [172, 173]. However, there are no reports about the crosstalk between H3K36me3 and repressive marks such as H3K9me and H3K27me, so whether the loss of demethylation of H3K9 and H3K27 affects the levels of H3K36me3 during transcription recovery is unclear.

Dysregulation of histone methylation marks plays a vital role in various ageing‐related disorders. In Alzheimer's disease, H3K9me3 is increased in the temporal cortex and hippocampus, involved in the functions of synaptic plasticity. This causes heterochromatin formation and silencing of gene expression and transcriptional repression [174]. Similarly, altered H3K27me3 marks can contribute to uncontrolled cell proliferation and metastasis, leading to cancer. Hypermethylation of H3K27me3 is observed in colorectal cancer, pancreatic cancer and prostate cancer [175]. H3K4me3 is increased on genes that determine haematopoietic stem cell (HSC) identity, affecting the functions of HSC during ageing [176]. Sarcopenia, an age‐related disorder causing a decline in muscle mass, is found to have altered changes in H3K4me3 [177]. Moreover, H3K4me and its role in regulating lifespan are well conserved among species. Loss of H3K4me3 can extend lifespan in a germline‐dependent manner in *C. elegans*. In mice, caloric restriction reduces H3K4me3 at the promoters of inflammatory genes, increasing its health span. Inhibition of H3K4 methylation also extends lifespan in yeast [178]. The above studies, highlighting the dynamics of histone methylation marks and their impact in ageing and longevity, show how these chromatin marks can be exploited to control organism ageing and functional decline in the DDR.

## Histone ubiquitination

Histone ubiquitination represents a distinct form of post‐translational modification, characterized by the attachment of ubiquitin, a 76 amino acid protein, to lysine residues on the target protein via an isopeptide bond [179]. This modification is regulated by three major ubiquitin enzymes: E1 (ubiquitin‐activating enzyme), E2 (ubiquitin‐conjugating enzymes) and E3 (ubiquitin ligases). E1 catalyses ATP‐dependent ubiquitin activation and creates a thioester bond between the ubiquitin C terminus and the catalytic cysteine on E1. Ubiquitin is then transferred to the catalytic cysteine on E2 and deposited to the target protein by E3 [180]. Protein ubiquitination is majorly known for its role during proteasome‐mediated degradation of targeted proteins. However, histone ubiquitination (mainly mono‐ubiquitination) is involved in the DDR primarily in a proteasome‐independent manner. Deubiquitination is achieved through the activity of deubiquitinating enzymes (DUBs) that interact with the ubiquitinated proteins and act as proteases to break the isopeptide bond binding ubiquitin to the target protein, thereby regulating biochemical activities on the chromatin.

### Histone ubiquitination in the DDR

Histones are the most abundant proteins that are ubiquitinated, majorly on the lysine residues of H2A, H2B and H4. Upon DNA damage, RNF8, an E3 ubiquitin ligase, is recruited to the damage sites by ATM phosphorylation and ubiquitinates linker histone H1 [181]. Later, this initial ubiquitination causes the recruitment of RNF168 E3 ligase, which in turn binds to the K63‐linked ubiquitin chains and catalyses mono‐ubiquitination of H2AK13 and K15. These modifications can recruit repair proteins such as 53BP1 (favours NHEJ) and the BRCA1‐A complex (favours HR) [182]. Defects in the H2AK13 and K15 ubiquitination lead to the loss of genome integrity and can cause an immunodeficiency syndrome called the RIDDLE syndrome [183]. RNF8 and RNF168 can also ubiquitinate H2AK127, which recruits SMARCAD1 in an ATP‐dependent manner and promotes DNA end resection, favouring HR [184].

DNA damage‐induced ATM activation also recruits the PRC1 complex consisting of the RING1B E3 ligase, that can ubiquitinate H2A on K119. These modifications are important for condensing the chromatin and preventing the binding of transcription factors, repressing transcription [185]. In addition, H3 and H4 ubiquitination mediated by CUL4‐DDB‐ROC1 is present in UV‐damaged DNA, recruiting XPC‐RAD23Bthat repair thymine dimers stabilizing the genome [186].

After the repair process is complete, the BRCC36 subunit of the BRCA1‐A complex acts as a K63 chain‐specific DUB to remove the ubiquitin groups from the chromatin [187].

In summary, histone ubiquitination is vital for remodelling the chromatin, regulating transcription, recruiting repair machinery and deciding repair pathway choice during the DDR.

### Histone ubiquitination in transcriptional regulation during the DDR

DNA damage can cause an imbalance of transcription and repair machinery. At DSB sites, H2AK13 and H2AK15 ubiquitination can recruit the PRC1 subunit RING1B that further ubiquitinates H2AK119. This mark can stabilize the silenced chromatin to prevent inefficient repair by recruiting PRC2 to deposit H3K27me3, a repressive transcription mark [188, 189].

Another ubiquitination mark, H2BK120, is deposited by RNF20, and plays a role in activating transcription during the DDR. Upon DNA damage, H2BK120ub1 opens the chromatin and recruits NHEJ and HR repair factors such as XRCC4, BRCA1 and RAD51, facilitating DNA repair [190]. It can promote the accumulation of the SPT16 subunit of the FACT complex, allowing RNA Pol II to move across gene bodies, promoting transcription of genes involved in DNA repair and cell cycle regulation [191]. Furthermore, it can stimulate the DOT1L methyltransferase enzyme, that methylates H3K79 and activates transcription [159, 192]. Evidence suggests that, upon DNA damage, H2BK120 ubiquitination cannot contribute to genome stability on its own, but that it recruits the FACT complex facilitating gene transcription and promoting Pol II‐mediated transcription elongation of SSRP1‐activated genes (which are subunits of the FACT complex) in mouse embryonic stem cells and yeast cells [193]. Studies also show that it can recruit the H3K4me3 enzyme COMPASS complex by Cps35, indicating the presence of crosstalk between H2B ubiquitination and transcriptionally active marks [194]. Further studies can be performed to investigate the role of Cps35 and the FACT complex with other histone modifications and understanding its common function in the transcription process. Ubiquitination on H2BK123 in yeast and mammalian cells is fundamental for DNA damage checkpoint activation. The absence of this mark inhibits RAD9 phosphorylation, allowing DNA repair by halting cell cycle progression and directly influencing RNA Pol II transcription [195].

To restore transcription after DNA repair, USP16, a DUB enzyme, removes the H2AK119ub mark and displaces the PRC components, thus reversing the chromatin repressive state and recovering transcription elongation [196].

Reports show that dysregulated histone ubiquitination in response to DSBs leads to cellular senescence, impaired proteostasis, ageing and neurodegenerative disorders [197]. A study shows that elevated levels of H2A ubiquitination can cause accelerated neurodegeneration and Alzheimer's disease [197]. Reduction of H2Aub increases the adult lifespan in *Drosophila melanogaster* and is evolutionarily conserved in mice, monkeys and humans [198]. This identifies H2Aub as a universal biomarker of ageing, and its interaction with H3K4 and H3K79 methylations can be investigated *in vivo*.

## Conclusion and future perspective

Transcriptional regulation in response to DNA damage is a highly coordinated process that can be divided into three steps: (1) rapid transcriptional repression at both local and global levels, (2) selective activation of DDR genes, and (3) eventual transcriptional recovery (Table 1). While the involvement of specific histone modifications in each phase has been delineated above, the precise mechanisms governing their dynamic crosstalk remain incompletely understood. A critical unresolved question is how transcription transitions through ‘access‐repair‐restore’ [199] chromatin states in a spatiotemporally controlled manner following DNA damage. For instance, both transcriptional repressive (e.g., H3K9 methylation) and activating (e.g., H3K4 methylation) marks play roles during the DDR. However, the way in which these two opposing marks interact with each other to influence the transcriptional state remains unclear. Key unknowns include how PTM‐modifying enzymes are recruited in response to TBLs and how they coordinate with the transcriptional machinery to regulate gene expression.

Furthermore, current technical limitations hinder the mapping of DNA lesions within high‐order chromatin structures, leaving a gap in our understanding of how chromatin architecture influences transcriptional regulation during the DDR. Another intriguing, yet underexplored aspect is the heritable nature of chromatin modifications. While histone marks are transiently altered during DDR, emerging evidence suggests they may contribute to an ‘epigenetic memory’ of cellular stress, [200] potentially influencing longevity and cellular fitness. Whether these modifications exert long‐term or even transgenerational effects remains an open question. Elucidating these mechanisms could uncover novel therapeutic strategies to mitigate the long‐term consequences of DNA damage.

Given the central role of DNA damage in ageing and cancer, histone modifications present a promising target for therapeutic intervention. For example, SPR5 (H3K4me2 demethylase) inhibition has the potential to modulate lifespan and transcriptional recovery upon UV‐induced damage in a model organism [163]. However, further study is needed to optimize their application—including timing, dosage and tissue specificity—while minimizing off‐target effects. A deeper understanding of histone modification dynamics during the DDR will not only advance fundamental biology but also pave the way for innovative treatments for age‐related diseases and cancer.

**Table 1:** List of histone modifications and their functions in response to DNA damage to regulate transcription.

Histone	Amino acid residue	Modification	Function after DNA damage	Function in transcription upon DNA damage
H2A	Tyrosine 142	Phosphorylation	Recruits RAD51 for transcription‐coupled HR	Transcription initiation
H2AX	Serine 139	Phosphorylation	Forms MRN complex and initiates DNA repair	Transcription initiation and elongation
H2A	Lysine 123	Ubiquitination	Phosphorylates RAD9	Regulates transcription elongation
H2A	Lysine 119	Ubiquitination	Releases RNA pol II	Transcription pause‐release
H2A	Lysine 15	Ubiquitination	Recruits 53BP1	Transcription stalling and represses initiation and elongation
H2A	Lysine 13	Ubiquitination	Recruits 53BP1	Transcription stalling and represses initiation and elongation
H2B	Lysine 120	Ubiquitination	Recruits FACT complex	Transcription elongation
H3	Lysine 122	Succinylation	Activates transcription and deposits at TSS	Transcription initiation
H3	Lysine 56	Acetylation	Can promote rapid nucleosome displacement	Transcription initiation
H3	Lysine 36	Methylation	Recruits YKU70 and maintains HR	Transcription elongation and recovery
H3	Lysine 27	Acetylation	Regulates levels of sirtuins and maintains transcription levels	Transcription initiation, elongation and recovery
H3	Lysine 18	Acetylation	Downregulates 53BP1	Transcription initiation and recovery
H3	Lysine 14	Acetylation	Retention of RSC and regulates DNA repair	Transcription initiation and elongation
H3	Threonine 11	Phosphorylation	Regulate gene expression	Transcription initiation
H3	Serine 10	Phosphorylation	Activates transcription	Transcription initiation and recovery of elongation
H3	Lysine 9	Acetylation	Can promote NHEJ by upregulating expression of DNA repair factors	Transcription initiation
H3	Lysine 9	Methylation	Regulates expression of DNA repair factors	Transcriptional silencing or repression
H3	Lysine 4	Methylation	Recruits yKu proteins and promotes NHEJ	Transcription initiation and elongation and recovery
H4	Lysine 16	Acetylation	NHEJ and HR repair	Transcription initiation and elongation
H4	Lysine 12	Acetylation	Recruit BRD2 to DSBs for repair	Transcription elongation
H4	Lysine 5	Acetylation	Reassembly of chromatin after DNA repair	Transcription regulation
H4	Serine 1	Phosphorylation	Re‐ligation of strands in NHEJ pathway	Transcription elongation

## Author contributions

AJK wrote the initial draft of the manuscript. SW supervised and revised the final version of the manuscript. All authors read and approved the submitted version.

## References

[1] Mariño‐Ramírez L , Kann MG , Shoemaker BA and Landsman D (2005) Histone structure and nucleosome stability. Expert Rev Proteomics 2, 719–729. 16209651 10.1586/14789450.2.5.719 PMC1831843

[2] Kujirai T and Kurumizaka H (2020) Transcription through the nucleosome. Curr Opin Struct Biol 61, 42–49. 31790919 10.1016/j.sbi.2019.10.007

[3] Petesch SJ and Lis JT (2008) Rapid, transcription‐independent loss of nucleosomes over a large chromatin domain at Hsp70 loci. Cell 134, 74–84. 18614012 10.1016/j.cell.2008.05.029 PMC2527511

[4] Fenley AT , Anandakrishnan R , Kidane YH and Onufriev AV (2018) Modulation of nucleosomal DNA accessibility via charge‐altering post‐translational modifications in histone core. Epigenetics Chromatin 11, 11. 29548294 10.1186/s13072-018-0181-5 PMC5856334

[5] Bannister AJ and Kouzarides T (2011) Regulation of chromatin by histone modifications. Cell Res 21, 381–395. 21321607 10.1038/cr.2011.22 PMC3193420

[6] Malik S and Roeder RG (2023) Regulation of the RNA polymerase II pre‐initiation complex by its associated coactivators. Nat Rev Genet 24, 767–782. 37532915 10.1038/s41576-023-00630-9 PMC11088444

[7] Gamarra N and Narlikar GJ (2021) Collaboration through chromatin: motors of transcription and chromatin structure. J Mol Biol 433, 166876. 33556407 10.1016/j.jmb.2021.166876 PMC8989640

[8] Winter GE , Mayer A , Buckley DL , Erb MA , Roderick JE , Vittori S , Reyes JM , di Iulio J , Souza A , Ott CJ *et al*. (2017) BET Bromodomain proteins function as master transcription elongation factors independent of CDK9 recruitment. Mol Cell 67, 5–18. 28673542 10.1016/j.molcel.2017.06.004 PMC5663500

[9] Mohamed AA , Vazquez Nunez R and Vos SM (2022) Structural advances in transcription elongation. Curr Opin Struct Biol 75, 102422. 35816930 10.1016/j.sbi.2022.102422 PMC9398977

[10] Bintu L , Ishibashi T , Dangkulwanich M , Wu Y‐Y , Lubkowska L , Kashlev M and Bustamante C (2012) Nucleosomal elements that control the topography of the barrier to transcription. Cell 151, 738–749. 23141536 10.1016/j.cell.2012.10.009 PMC3508686

[11] Kireeva ML , Walter W , Tchernajenko V , Bondarenko V , Kashlev M and Studitsky VM (2002) Nucleosome remodeling induced by RNA polymerase II: loss of the H2A/H2B dimer during transcription. Mol Cell 9, 541–552. 11931762 10.1016/s1097-2765(02)00472-0

[12] Žumer K , Ochmann M , Aljahani A , Zheenbekova A , Devadas A , Maier KC , Rus P , Neef U , Oudelaar AM and Cramer P (2024) FACT maintains chromatin architecture and thereby stimulates RNA polymerase II pausing during transcription in vivo. Mol Cell 84, 2053–2069. 38810649 10.1016/j.molcel.2024.05.003

[13] Tufegdžić Vidaković A , Mitter R , Kelly GP , Neumann M , Harreman M , Rodríguez‐Martínez M , Herlihy A , Weems JC , Boeing S , Encheva V *et al*. (2020) Regulation of the RNAPII pool is integral to the DNA damage response. Cell 180, 1245–1261. 32142654 10.1016/j.cell.2020.02.009 PMC7103762

[14] Hanawalt PC (2000) DNA repair. The bases for Cockayne syndrome. Nature 405, 415–416. 10839526 10.1038/35013197

[15] Gupta S , You P , SenGupta T , Nilsen H and Sharma K (2021) Crosstalk between different DNA repair pathways contributes to neurodegenerative diseases. Biology 10, 163. 33669593 10.3390/biology10020163 PMC7922961

[16] Black JO (2016) Xeroderma Pigmentosum. Head Neck Pathol 10, 139–144. 26975629 10.1007/s12105-016-0707-8 PMC4838978

[17] Hashimoto S and Egly JM (2009) Trichothiodystrophy view from the molecular basis of DNA repair/transcription factor TFIIH. Hum Mol Genet 18, R224–R230. 19808800 10.1093/hmg/ddp390

[18] Hassa PO and Hottiger MO (2002) The functional role of poly(ADP‐ribose)polymerase 1 as novel coactivator of NF‐κB in inflammatory disorders. CMLS, Cell Mol Life Sci 59, 1534–1553. 12440774 10.1007/s00018-002-8527-2 PMC11337477

[19] Xu J , Lahiri I , Wang W , Wier A , Cianfrocco MA , Chong J , Hare AA , Dervan PB , DiMaio F , Leschziner AE *et al*. (2017) Structural basis for the initiation of eukaryotic transcription‐coupled DNA repair. Nature 551, 653–657. 29168508 10.1038/nature24658 PMC5907806

[20] Fischer ES , Scrima A , Böhm K , Matsumoto S , Lingaraju GM , Faty M , Yasuda T , Cavadini S , Wakasugi M , Hanaoka F *et al*. (2011) The molecular basis of CRL4DDB2/CSA ubiquitin ligase architecture, targeting, and activation. Cell 147, 1024–1039. 22118460 10.1016/j.cell.2011.10.035

[21] Ramadhin AR , Lee S‐H , Zhou D , Salmazo A , Gonzalo‐Hansen C , van Sluis M , Blom CMA , Janssens RC , Raams A , Dekkers D *et al*. (2024) STK19 drives transcription‐coupled repair by stimulating repair complex stability, RNA pol II ubiquitylation, and TFIIH recruitment. Mol Cell 84, 4740–4757. 39547223 10.1016/j.molcel.2024.10.030

[22] Liebau RC , Waters C , Ahmed A , Soni RK and Gautier J (2024) UVSSA facilitates transcription‐coupled repair of DNA interstrand crosslinks. DNA Repair 143, 103771. 39383571 10.1016/j.dnarep.2024.103771

[23] Xu J , Chong J and Wang D (2021) Strand‐specific effect of Rad26 and TFIIS in rescuing transcriptional arrest by CAG trinucleotide repeat slip‐outs. Nucleic Acids Res 49, 7618–7627. 34197619 10.1093/nar/gkab573 PMC8287942

[24] Compe E and Egly J‐M (2012) TFIIH: when transcription met DNA repair. Nat Rev Mol Cell Biol 13, 343–354. 22572993 10.1038/nrm3350

[25] Mason PB and Struhl K (2003) The FACT complex travels with elongating RNA polymerase II and is important for the fidelity of transcriptional initiation in vivo. Mol Cell Biol 23, 8323–8333. 14585989 10.1128/MCB.23.22.8323-8333.2003 PMC262413

[26] Wienholz F , Zhou D , Turkyilmaz Y , Schwertman P , Tresini M , Pines A , van Toorn M , Bezstarosti K , Demmers JAA and Marteijn JA (2019) FACT subunit Spt16 controls UVSSA recruitment to lesion‐stalled RNA pol II and stimulates TC‐NER. Nucleic Acids Res 47, 4011–4025. 30715484 10.1093/nar/gkz055 PMC6486547

[27] van Sluis M , Yu Q , van der Woude M , Gonzalo‐Hansen C , Dealy SC , Janssens RC , Somsen HB , Ramadhin AR , Dekkers DHW , Wienecke HL *et al*. (2024) Transcription‐coupled DNA–protein crosslink repair by CSB and CRL4CSA‐mediated degradation. Nat Cell Biol 26, 770–783. 38600236 10.1038/s41556-024-01394-y PMC11098752

[28] Carnie CJ , Acampora AC , Bader AS , Erdenebat C , Zhao S , Bitensky E , van den Heuvel D , Parnas A , Gupta V , D'Alessandro G *et al*. (2024) Transcription‐coupled repair of DNA–protein cross‐links depends on CSA and CSB. Nat Cell Biol 26, 797–810. 38600235 10.1038/s41556-024-01391-1 PMC11098753

[29] Carnie CJ , Jackson SP and Stingele J (2025) Transcription‐coupled repair of DNA‐protein crosslinks. Trends Cell Biol 35, 316–329. 39617652 10.1016/j.tcb.2024.11.003

[30] Li L (2024) Transcription reprogramming and endogenous DNA damage. DNA Repair 142, 103754. 39232366 10.1016/j.dnarep.2024.103754

[31] Vaz B , Popovic M and Ramadan K (2017) DNA–protein crosslink proteolysis repair. Trends Biochem Sci 42, 483–495. 28416269 10.1016/j.tibs.2017.03.005

[32] Li F , Zafar A , Luo L , Denning AM , Gu J , Bennett A , Yuan F and Zhang Y (2023) R‐loops in genome instability and cancer. Cancers 15, 4986. 37894353 10.3390/cancers15204986 PMC10605827

[33] Williams AB and Schumacher B (2016) p53 in the DNA‐damage‐repair process. Cold Spring Harb Perspect Med 6, a026070. 27048304 10.1101/cshperspect.a026070 PMC4852800

[34] Lin Y , Qiu T , Wei G , Que Y , Wang W , Kong Y , Xie T and Chen X (2022) Role of histone post‐translational modifications in inflammatory diseases. Front Immunol 13, 852272. 35280995 10.3389/fimmu.2022.852272 PMC8908311

[35] Tamagawa H , Oshima T , Shiozawa M , Morinaga S , Nakamura Y , Yoshihara M , Sakuma Y , Kameda Y , Akaike M , Masuda M *et al*. (2012) The global histone modification pattern correlates with overall survival in metachronous liver metastasis of colorectal cancer. Oncol Rep 27, 637–642. 22076537 10.3892/or.2011.1547

[36] Yang Y , Zhang M and Wang Y (2022) The roles of histone modifications in tumorigenesis and associated inhibitors in cancer therapy. J Natl Cancer Cent 2, 277–290. 39036551 10.1016/j.jncc.2022.09.002 PMC11256729

[37] Zandarashvili L , Langelier M‐F , Velagapudi UK , Hancock MA , Steffen JD , Billur R , Hannan ZM , Wicks AJ , Krastev DB , Pettitt SJ *et al*. (2020) Structural basis for allosteric PARP‐1 retention on DNA breaks. Science 368, eaax6367. 32241924 10.1126/science.aax6367 PMC7347020

[38] van Beek L , McClay É , Patel S , Schimpl M , Spagnolo L and Maia de Oliveira T (2021) PARP power: a structural perspective on PARP1, PARP2, and PARP3 in DNA damage repair and nucleosome Remodelling. Int J Mol Sci 22, 5112. 34066057 10.3390/ijms22105112 PMC8150716

[39] Kouyama K , Mayanagi K , Nakae S , Nishi Y , Miwa M and Shirai T (2019) Single‐particle analysis of full‐length human poly(ADP‐ribose) polymerase 1. Biophys Physicobiol 16, 59–67. 30923663 10.2142/biophysico.16.0_59 PMC6435018

[40] Adamson B , Smogorzewska A , Sigoillot FD , King RW and Elledge SJ (2012) A genome‐wide homologous recombination screen identifies the RNA‐binding protein RBMX as a component of the DNA‐damage response. Nat Cell Biol 14, 318–328. 22344029 10.1038/ncb2426 PMC3290715

[41] Spiegel JO , Van Houten B and Durrant JD (2021) PARP1: structural insights and pharmacological targets for inhibition. DNA Repair 103, 103125. 33940558 10.1016/j.dnarep.2021.103125 PMC8206044

[42] Hoch NC , Hanzlikova H , Rulten SL , Tétreault M , Komulainen E , Ju L , Hornyak P , Zeng Z , Gittens W , Rey SA *et al*. (2017) XRCC1 mutation is associated with PARP1 hyperactivation and cerebellar ataxia. Nature 541, 87–91. 28002403 10.1038/nature20790 PMC5218588

[43] Maltseva EA , Rechkunova NI , Sukhanova MV and Lavrik OI (2015) Poly(ADP‐ribose) polymerase 1 modulates interaction of the nucleotide excision repair factor XPC‐RAD23B with DNA via poly(ADP‐ribosyl)ation. J Biol Chem 290, 21811–21820. 26170451 10.1074/jbc.M115.646638 PMC4571937

[44] Bryant HE , Petermann E , Schultz N , Jemth A , Loseva O , Issaeva N , Johansson F , Fernandez S , McGlynn P and Helleday T (2009) PARP is activated at stalled forks to mediate Mre11‐dependent replication restart and recombination. EMBO J 28, 2601–2615. 19629035 10.1038/emboj.2009.206 PMC2738702

[45] Caron M‐C , Sharma AK , O'Sullivan J , Myler LR , Ferreira MT , Rodrigue A , Coulombe Y , Ethier C , Gagné J‐P , Langelier M‐F *et al*. (2019) Poly(ADP‐ribose) polymerase‐1 antagonizes DNA resection at double‐strand breaks. Nat Commun 10, 2954. 31273204 10.1038/s41467-019-10741-9 PMC6609622

[46] McGurk L , Rifai OM and Bonini NM (2019) Poly(ADP‐ribosylation) in age‐related neurological disease. Trends Genet 35, 601–613. 31182245 10.1016/j.tig.2019.05.004 PMC6625889

[47] Gibbs‐Seymour I , Fontana P , Rack JGM and Ahel I (2016) HPF1/C4orf27 is a PARP‐1‐interacting protein that regulates PARP‐1 ADP‐Ribosylation activity. Mol Cell 62, 432–442. 27067600 10.1016/j.molcel.2016.03.008 PMC4858568

[48] Adamowicz M , Hailstone R , Demin AA , Komulainen E , Hanzlikova H , Brazina J , Gautam A , Wells SE and Caldecott KW (2021) XRCC1 protects transcription from toxic PARP1 activity during DNA base excision repair. Nat Cell Biol 23, 1287–1298. 34811483 10.1038/s41556-021-00792-w PMC8683375

[49] Reber JM , Božić‐Petković J , Lippmann M , Mazzardo M , Dilger A , Warmers R , Bürkle A and Mangerich A (2023) PARP1 and XRCC1 exhibit a reciprocal relationship in genotoxic stress response. Cell Biol Toxicol 39, 345–364. 35778544 10.1007/s10565-022-09739-9 PMC10042965

[50] Lee S‐G , Kim N , Kim S‐M , Park IB , Kim H , Kim S , Kim B‐G , Hwang JM , Baek I‐J , Gartner A *et al*. (2020) Ewing sarcoma protein promotes dissociation of poly(ADP‐ribose) polymerase 1 from chromatin. EMBO J Rep 21, e48676. 10.15252/embr.201948676 PMC7645264 33006225

[51] Alirzayeva H , Loureiro R , Koyuncu S , Hommen F , Nabawi Y , Zhang WH , Dao TTP , Wehrmann M , Lee HJ and Vilchez D (2024) ALS‐FUS mutations cause abnormal PARylation and histone H1.2 interaction, leading to pathological changes. Cell Rep 43, 114626. 39167487 10.1016/j.celrep.2024.114626

[52] Andronikou C and Rottenberg S (2021) Studying PAR‐dependent chromatin remodeling to tackle PARPi resistance. Trends Mol Med 27, 630–642. 34030964 10.1016/j.molmed.2021.04.010

[53] Páhi ZG , Borsos BN , Pantazi V , Ujfaludi Z and Pankotai T (2020) PARylation during transcription: insights into the fine‐tuning mechanism and regulation. Cancers 12, 183. 31940791 10.3390/cancers12010183 PMC7017041

[54] Yang G , Chen Y , Wu J , Chen S‐H , Liu X , Singh AK and Yu X (2020) Poly(ADP‐ribosyl)ation mediates early phase histone eviction at DNA lesions. Nucleic Acids Res 48, 3001–3013. 31965183 10.1093/nar/gkaa022 PMC7102957

[55] Chou DM , Adamson B , Dephoure NE , Tan X , Nottke AC , Hurov KE , Gygi SP , Colaiácovo MP and Elledge SJ (2010) A chromatin localization screen reveals poly (ADP ribose)‐regulated recruitment of the repressive polycomb and NuRD complexes to sites of DNA damage. Proc Natl Acad Sci 107, 18475–18480. 20937877 10.1073/pnas.1012946107 PMC2972950

[56] Langelier M‐F and Pascal JM (2013) PARP‐1 mechanism for coupling DNA damage detection to poly(ADP‐ribose) synthesis. Curr Opin Struct Biol 23, 134–143. 23333033 10.1016/j.sbi.2013.01.003 PMC3572337

[57] Fu H , Liu R , Jia Z , Li R , Zhu F , Zhu W , Shao Y , Jin Y , Xue Y , Huang J *et al*. (2022) Poly(ADP‐ribosylation) of P‐TEFb by PARP1 disrupts phase separation to inhibit global transcription upon DNA damage. Nat Cell Biol 24, 513–525. 35393539 10.1038/s41556-022-00872-5 PMC9035116

[58] Li X , Liu L , Yang S , Song N , Zhou X , Gao J , Yu N , Shan L , Wang Q , Liang J *et al*. (2014) Histone demethylase KDM5B is a key regulator of genome stability. Proc Natl Acad Sci USA 111, 7096–7101. 24778210 10.1073/pnas.1324036111 PMC4024858

[59] Weaver AN and Yang ES (2013) Beyond DNA repair: additional functions of PARP‐1 in cancer. Front Oncol 3, 290. 24350055 10.3389/fonc.2013.00290 PMC3841914

[60] Khoury‐Haddad H , Guttmann‐Raviv N , Ipenberg I , Huggins D , Jeyasekharan AD and Ayoub N (2014) PARP1‐dependent recruitment of KDM4D histone demethylase to DNA damage sites promotes double‐strand break repair. Proc Natl Acad Sci USA 111, E728–E737. 24550317 10.1073/pnas.1317585111 PMC3932863

[61] Savchenko VL (2024) Poly‐ADP‐ribosylation of KDM4D induces transcription in the hippocampus and amygdala.

[62] Young LC , McDonald DW and Hendzel MJ (2013) Kdm4b histone demethylase is a DNA damage response protein and confers a survival advantage following γ‐irradiation. J Biol Chem 288, 21376–21388. 23744078 10.1074/jbc.M113.491514 PMC3774405

[63] Izhar L , Adamson B , Ciccia A , Lewis J , Pontano‐Vaites L , Leng Y , Liang AC , Westbrook TF , Harper JW and Elledge SJ (2015) A systematic analysis of factors localized to damaged chromatin reveals PARP‐dependent recruitment of transcription factors. Cell Rep 11, 1486–1500. 26004182 10.1016/j.celrep.2015.04.053 PMC4464939

[64] Hunter JE , Willmore E , Irving JAE , Hostomsky Z , Veuger SJ and Durkacz BW (2012) NF‐κB mediates radio‐sensitization by the PARP‐1 inhibitor, AG‐014699. Oncogene 31, 251–264. 21706052 10.1038/onc.2011.229 PMC3191117

[65] Simbulan‐Rosenthal CM , Rosenthal DS , Luo R , Samara R , Espinoza LA , Hassa PO , Hottiger MO and Smulson ME (2003) PARP‐1 binds E2F‐1 independently of its DNA binding and catalytic domains, and acts as a novel coactivator of E2F‐1‐mediated transcription during re‐entry of quiescent cells into S phase. Oncogene 22, 8460–8471. 14627987 10.1038/sj.onc.1206897

[66] Iglesias P , Seoane M , Golán I , Castro‐Piedras I , Fraga M , Arce VM and Costoya JA (2020) PARP1 deficiency reduces tumour growth by decreasing E2F1 hyperactivation: a novel mechanism in the treatment of cancer. Cancers 12, 2907. 33050515 10.3390/cancers12102907 PMC7599842

[67] Biswas AK and Johnson DG (2012) Transcriptional and nontranscriptional functions of E2F1 in response to DNA damage. Cancer Res 72, 13–17. 22180494 10.1158/0008-5472.CAN-11-2196 PMC3563329

[68] Ahel I , Ahel D , Matsusaka T , Clark AJ , Pines J , Boulton SJ and West SC (2008) Poly(ADP‐ribose)‐binding zinc finger motifs in DNA repair/checkpoint proteins. Nature 451, 81–85. 18172500 10.1038/nature06420

[69] Wang Y‐H , Ho TLF , Hariharan A , Goh HC , Wong YL , Verkaik NS , Lee MY , Tam WL , van Gent DC , Venkitaraman AR *et al*. (2022) Rapid recruitment of p53 to DNA damage sites directs DNA repair choice and integrity. Proc Natl Acad Sci 119, e2113233119. 35235448 10.1073/pnas.2113233119 PMC8915893

[70] Gajewski S and Hartwig A (2020) PARP1 is required for ATM‐mediated p53 activation and p53‐mediated gene expression after ionizing radiation. Chem Res Toxicol 33, 1933–1940. 32551582 10.1021/acs.chemrestox.0c00130

[71] Chao C , Saito S , Kang J , Anderson CW , Appella E and Xu Y (2000) p53 transcriptional activity is essential for p53‐dependent apoptosis following DNA damage. EMBO J 19, 4967–4975. 10990460 10.1093/emboj/19.18.4967 PMC314218

[72] Sharma D , De Falco L , Padavattan S , Rao C , Geifman‐Shochat S , Liu C‐F and Davey CA (2019) PARP1 exhibits enhanced association and catalytic efficiency with γH2A.X‐nucleosome. Nat Commun 10, 5751. 31848352 10.1038/s41467-019-13641-0 PMC6917767

[73] Tyagi W and Das S (2024) Temporal regulation of acetylation status determines PARP1 role in DNA damage response and metabolic homeostasis. Sci Adv 10, eado7720. 39423262 10.1126/sciadv.ado7720 PMC11488539

[74] Mao K and Zhang G (2022) The role of PARP1 in neurodegenerative diseases and aging. FEBS J 289, 2013–2024. 33460497 10.1111/febs.15716

[75] Prokhorova E , Agnew T , Wondisford AR , Tellier M , Kaminski N , Beijer D , Holder J , Groslambert J , Suskiewicz MJ , Zhu K *et al*. (2021) Unrestrained poly‐ADP‐ribosylation provides insights into chromatin regulation and human disease. Mol Cell 81, 2640–2655. 34019811 10.1016/j.molcel.2021.04.028 PMC8221567

[76] DaRosa PA , Wang Z , Jiang X , Pruneda JN , Cong F , Klevit RE and Xu W (2015) Allosteric activation of the RNF146 ubiquitin ligase by a poly(ADP‐ribosyl)ation signal. Nature 517, 223–226. 25327252 10.1038/nature13826 PMC4289021

[77] Gibson BA , Zhang Y , Jiang H , Hussey KM , Shrimp JH , Lin H , Schwede F , Yu Y and Kraus WL (2016) Chemical genetic discovery of PARP targets reveals a role for PARP‐1 in transcription elongation. Science 353, 45–50. 27256882 10.1126/science.aaf7865 PMC5540732

[78] Bondar D and Karpichev Y (2024) Poly(ADP‐ribose) polymerase (PARP) inhibitors for cancer therapy: advances, challenges, and future directions. Biomolecules 14, 1269. 39456202 10.3390/biom14101269 PMC11506039

[79] Gong P , Guo Z , Wang S , Gao S and Cao Q (2025) Histone phosphorylation in DNA damage response. Int J Mol Sci 26, 2405. 40141048 10.3390/ijms26062405 PMC11941871

[80] Nam SM and Cho K‐O (2017) Chapter 12 – the role of epigenetics in the pathophysiology of epilepsy. In Neuropsychiatric Disorders and Epigenetics ( Yasui DH , Peedicayil J and Grayson DR , eds), pp. 233–260. Academic Press, Boston.

[81] Rogakou EP , Pilch DR , Orr AH , Ivanova VS and Bonner WM (1998) DNA double‐stranded breaks induce histone H2AX phosphorylation on serine 139. J Biol Chem 273, 5858–5868. 9488723 10.1074/jbc.273.10.5858

[82] Phan LM and Rezaeian A‐H (2021) ATM: Main features, signaling pathways, and its diverse roles in DNA damage response, tumor suppression, and cancer development. Genes 12, 845. 34070860 10.3390/genes12060845 PMC8228802

[83] Ji J‐H , Min S , Chae S , Ha G‐H , Kim Y , Park Y‐J , Lee C‐W and Cho H (2019) De novo phosphorylation of H2AX by WSTF regulates transcription‐coupled homologous recombination repair. Nucleic Acids Res 47, 6299–6314. 31045206 10.1093/nar/gkz309 PMC6614800

[84] Kobayashi J , Antoccia A , Tauchi H , Matsuura S and Komatsu K (2004) NBS1 and its functional role in the DNA damage response. DNA Repair 3, 855–861. 15279770 10.1016/j.dnarep.2004.03.023

[85] Salzano M , Sanz‐García M , Monsalve DM , Moura DS and Lazo PA (2015) VRK1 chromatin kinase phosphorylates H2AX and is required for foci formation induced by DNA damage. Epigenetics 10, 373–383. 25923214 10.1080/15592294.2015.1028708 PMC4623420

[86] Celeste A , Petersen S , Romanienko PJ , Fernandez‐Capetillo O , Chen HT , Sedelnikova OA , Reina‐San‐Martin B , Coppola V , Meffre E , Difilippantonio MJ *et al*. (2002) Genomic instability in mice lacking histone H2AX. Science 296, 922–927. 11934988 10.1126/science.1069398 PMC4721576

[87] He Z‐Y , Wang W‐Y , Hu W‐Y , Yang L , Li Y , Zhang W‐Y , Yang Y‐S , Liu S‐C , Zhang F‐L , Mei R *et al*. (2016) Gamma‐H2AX upregulation caused by Wip1 deficiency increases depression‐related cellular senescence in hippocampus. Sci Rep 6, 34558. 27686532 10.1038/srep34558 PMC5043360

[88] Moon S‐H , Nguyen T‐A , Darlington Y , Lu X and Donehower LA (2010) Dephosphorylation of γ‐H2AX by WIP1: an important homeostatic regulatory event in DNA repair and cell cycle control. Cell Cycle 9, 2092–2096. 20495376 10.4161/cc.9.11.11810 PMC3984036

[89] Krum SA , Dalugdugan EDLR , Miranda‐Carboni GA and Lane TF (2010) BRCA1 forms a functional complex with γ‐H2AX as a late response to genotoxic stress. J Nucleic Acids 2010, 801594. 20936109 10.4061/2010/801594 PMC2948912

[90] Utley RT , Lacoste N , Jobin‐Robitaille O , Allard S and Côté J (2005) Regulation of NuA4 histone acetyltransferase activity in transcription and DNA repair by phosphorylation of histone H4. Mol Cell Biol 25, 8179–8190. 16135807 10.1128/MCB.25.18.8179-8190.2005 PMC1234332

[91] Li J , Mahata B , Escobar M , Goell J , Wang K , Khemka P and Hilton IB (2021) Programmable human histone phosphorylation and gene activation using a CRISPR/Cas9‐based chromatin kinase. Nat Commun 12, 896. 33563994 10.1038/s41467-021-21188-2 PMC7873277

[92] Banerjee T and Chakravarti D (2011) A peek into the complex realm of histone phosphorylation. Mol Cell Biol 31, 4858–4873. 22006017 10.1128/MCB.05631-11 PMC3233023

[93] Burma S , Chen BP , Murphy M , Kurimasa A and Chen DJ (2001) ATM phosphorylates histone H2AX in response to DNA double‐strand breaks. J Biol Chem 276, 42462–42467. 11571274 10.1074/jbc.C100466200

[94] Day M , Oliver AW and Pearl LH (2021) Phosphorylation‐dependent assembly of DNA damage response systems and the central roles of TOPBP1. DNA Repair (Amst) 108, 103232. 34678589 10.1016/j.dnarep.2021.103232 PMC8651625

[95] Shanbhag NM , Rafalska‐Metcalf IU , Balane‐Bolivar C , Janicki SM and Greenberg RA (2010) ATM‐dependent chromatin changes silence transcription in cis to DNA double‐strand breaks. Cell 141, 970–981. 20550933 10.1016/j.cell.2010.04.038 PMC2920610

[96] Ketley RF , Battistini F , Alagia A , Mondielli C , Iehl F , Balikçi E , Huber KVM , Orozco M and Gullerova M (2022) DNA double‐strand break‐derived RNA drives TIRR/53BP1 complex dissociation. Cell Rep 41, 111526. 36288694 10.1016/j.celrep.2022.111526 PMC9638026

[97] Kovaříková AS , Legartová S , Krejčí J and Bártová E (2018) H3K9me3 and H4K20me3 represent the epigenetic landscape for 53BP1 binding to DNA lesions. Aging 10, 2585–2605. 30312172 10.18632/aging.101572 PMC6224238

[98] Pankotai T , Bonhomme C , Chen D and Soutoglou E (2012) DNAPKcs‐dependent arrest of RNA polymerase II transcription in the presence of DNA breaks. Nat Struct Mol Biol 19, 276–282. 22343725 10.1038/nsmb.2224

[99] Paquin KL and Howlett NG (2018) The histone DNA repair code: H4K20me2 makes its mark. Mol Cancer Res 16, 1335–1345. 29858375 10.1158/1541-7786.MCR-17-0688 PMC7083049

[100] Botuyan MV , Lee J , Ward IM , Kim J‐E , Thompson JR , Chen J and Mer G (2006) Structural basis for the methylation state‐specific recognition of histone H4‐K20 by 53BP1 and Crb2 in DNA repair. Cell 127, 1361–1373. 17190600 10.1016/j.cell.2006.10.043 PMC1804291

[101] Chowdhury D , Keogh M‐C , Ishii H , Peterson CL , Buratowski S and Lieberman J (2005) γ‐H2AX Dephosphorylation by protein phosphatase 2A facilitates DNA double‐Strand break repair. Mol Cell 20, 801–809. 16310392 10.1016/j.molcel.2005.10.003

[102] Komar D and Juszczynski P (2020) Rebelled epigenome: histone H3S10 phosphorylation and H3S10 kinases in cancer biology and therapy. Clin Epigenetics 12, 147. 33054831 10.1186/s13148-020-00941-2 PMC7556946

[103] Notani D (2024) Compaction of active chromatin domains and promoters by H3S10 phosphorylation during mitosis preserves interphase‐specific chromatin structure and function.

[104] Soloaga A , Thomson S , Wiggin GR , Rampersaud N , Dyson MH , Hazzalin CA , Mahadevan LC and Arthur JSC (2003) MSK2 and MSK1 mediate the mitogen‐ and stress‐induced phosphorylation of histone H3 and HMG‐14. EMBO J 22, 2788–2797. 12773393 10.1093/emboj/cdg273 PMC156769

[105] Zhu X , Li D , Zhang Z , Zhu W , Li W , Zhao J , Xing X , He Z , Wang S , Wang F *et al*. (2017) Persistent phosphorylation at specific H3 serine residues involved in chemical carcinogen‐induced cell transformation. Mol Carcinog 56, 1449–1460. 27996159 10.1002/mc.22605

[106] Suberbielle E , Sanchez PE , Kravitz AV , Wang X , Ho K , Eilertson K , Devidze N , Kreitzer AC and Mucke L (2013) Physiological brain activity causes DNA double strand breaks in neurons – exacerbation by amyloid‐β. Nat Neurosci 16, 613–621. 23525040 10.1038/nn.3356 PMC3637871

[107] Guo Z , Pei S , Si T , Li J , Jiang C , Li S and Zhao J (2015) Expression of the γ‐phosphorylated histone H2AX in gastric carcinoma and gastric precancerous lesions. Oncol Lett 9, 1790–1794. 25789044 10.3892/ol.2015.2896 PMC4356332

[108] He Y , Gong Y , Lin J , Chang DW , Gu J , Roth JA and Wu X (2013) Ionizing radiation‐induced γ‐H2AX activity in whole blood culture and the risk of lung cancer. Cancer Epidemiol Biomarkers Prev 22, 443–451. 23300022 10.1158/1055-9965.EPI-12-0794 PMC3601549

[109] Warters RL , Adamson PJ , Pond CD and Leachman SA (2005) Melanoma cells express elevated levels of phosphorylated histone H2AX foci. J Invest Dermatol 124, 807–817. 15816840 10.1111/j.0022-202X.2005.23674.x

[110] Yu T , MacPhail SH , Banáth JP , Klokov D and Olive PL (2006) Endogenous expression of phosphorylated histone H2AX in tumors in relation to DNA double‐strand breaks and genomic instability. DNA Repair (Amst) 5, 935–946. 16814620 10.1016/j.dnarep.2006.05.040

[111] Lee Y‐C , Yin TC , Chen Y‐T , Chai C‐Y , Wang JY , Liu M‐C , Lin Y‐C and Kan JY (2015) High expression of phospho‐H2AX predicts a poor prognosis in colorectal cancer. Anticancer Res 35, 2447–2453. 25862913

[112] Gorgoulis VG , Vassiliou L‐VF , Karakaidos P , Zacharatos P , Kotsinas A , Liloglou T , Venere M , Ditullio RA , Kastrinakis NG , Levy B *et al*. (2005) Activation of the DNA damage checkpoint and genomic instability in human precancerous lesions. Nature 434, 907–913. 15829965 10.1038/nature03485

[113] Nagelkerke A , van Kuijk SJA , Sweep FCGJ , Nagtegaal ID , Hoogerbrugge N , Martens JWM , Timmermans MA , van Laarhoven HWM , Bussink J and Span PN (2011) Constitutive expression of γ‐H2AX has prognostic relevance in triple negative breast cancer. Radiother Oncol 101, 39–45. 21840613 10.1016/j.radonc.2011.07.009

[114] Sawicka A and Seiser C (2012) Histone H3 phosphorylation – a versatile chromatin modification for different occasions. Biochimie 94, 2193–2201. 22564826 10.1016/j.biochi.2012.04.018 PMC3480636

[115] Kawashima S , Kawaguchi N , Taniguchi K , Tashiro K , Komura K , Tanaka T , Inomata Y , Imai Y , Tanaka R , Yamamoto M *et al*. (2020) γ‐H2AX as a potential indicator of radiosensitivity in colorectal cancer cells. Oncol Lett 20, 2331–2337. 32782550 10.3892/ol.2020.11788 PMC7400563

[116] Yang SX , Polley EC and Nguyen D (2017) Association of γH2AX at diagnosis with chemotherapy outcome in patients with breast cancer. Theranostics 7, 945–951. 28382166 10.7150/thno.19102 PMC5381256

[117] Dhar S , Gursoy‐Yuzugullu O , Parasuram R and Price BD (2017) The tale of a tail: histone H4 acetylation and the repair of DNA breaks. Philos Trans R Soc B: Biol Sci 372, 20160284. 10.1098/rstb.2016.0284 PMC5577462 28847821

[118] Görisch SM , Wachsmuth M , Tóth KF , Lichter P and Rippe K (2005) Histone acetylation increases chromatin accessibility. J Cell Sci 118, 5825–5834. 16317046 10.1242/jcs.02689

[119] Aricthota S , Rana PP and Haldar D (2022) Histone acetylation dynamics in repair of DNA double‐strand breaks. Front Genet 13, 926577. 36159966 10.3389/fgene.2022.926577 PMC9503837

[120] Ahmad S , Côté V , Cheng X , Bourriquen G , Sapountzi V , Altaf M and Côté J (2021) Antagonistic relationship of NuA4 with the non‐homologous end‐joining machinery at DNA damage sites. PLoS Genet 17, e1009816. 34543274 10.1371/journal.pgen.1009816 PMC8483352

[121] Noguchi C , Singh T , Ziegler MA , Peake JD , Khair L , Aza A , Nakamura TM and Noguchi E (2019) The NuA4 acetyltransferase and histone H4 acetylation promote replication recovery after topoisomerase I‐poisoning. Epigenetics Chromatin 12, 24. 30992049 10.1186/s13072-019-0271-z PMC6466672

[122] González‐Bermúdez L , Genescà A , Terradas M and Martín M (2022) Role of H4K16 acetylation in 53BP1 recruitment to double‐strand break sites in in vitro aged cells. Biogerontology 23, 499–514. 35851632 10.1007/s10522-022-09979-6 PMC9388460

[123] Kusch T , Florens L , Macdonald WH , Swanson SK , Glaser RL , Yates JR , Abmayr SM , Washburn MP and Workman JL (2004) Acetylation by Tip60 is required for selective histone variant exchange at DNA lesions. Science 306, 2084–2087. 15528408 10.1126/science.1103455

[124] Swift ML , Beishline K and Azizkhan‐Clifford J (2021) Sp1‐dependent recruitment of the histone acetylase p300 to DSBs facilitates chromatin remodeling and recruitment of the NHEJ repair factor Ku70. DNA Repair 105, 103171. 34252870 10.1016/j.dnarep.2021.103171

[125] Zhang A , Chen L , Ma L , Ding X , Tang S , Zhang A and Li J (2020) Role of H3K18ac‐regulated nucleotide excision repair‐related genes in arsenic‐induced DNA damage and repair of HaCaT cells. Hum Exp Toxicol 39, 1168–1177. 32031413 10.1177/0960327120903482

[126] Jacquet K , Fradet‐Turcotte A , Avvakumov N , Lambert J‐P , Roques C , Pandita RK , Paquet E , Herst P , Gingras A‐C , Pandita TK *et al*. (2016) The TIP60 complex regulates bivalent chromatin recognition by 53BP1 through direct H4K20me binding and H2AK15 acetylation. Mol Cell 62, 409–421. 27153538 10.1016/j.molcel.2016.03.031 PMC4887106

[127] Tjeertes JV , Miller KM and Jackson SP (2009) Screen for DNA‐damage‐responsive histone modifications identifies H3K9Ac and H3K56Ac in human cells. EMBO J 28, 1878–1889. 19407812 10.1038/emboj.2009.119 PMC2684025

[128] Miller KM , Tjeertes JV , Coates J , Legube G , Polo SE , Britton S and Jackson SP (2010) Human HDAC1 and HDAC2 function in the DNA‐damage response to promote DNA nonhomologous end‐joining. Nat Struct Mol Biol 17, 1144–1151. 20802485 10.1038/nsmb.1899 PMC3018776

[129] Duan M‐R and Smerdon MJ (2014) Histone H3 lysine 14 (H3K14) acetylation facilitates DNA repair in a positioned nucleosome by stabilizing the binding of the chromatin remodeler RSC (remodels structure of chromatin)*. J Biol Chem 289, 8353–8363. 24515106 10.1074/jbc.M113.540732 PMC3961661

[130] Cote JM , Kuo Y‐M , Henry RA , Scherman H , Krzizike DD and Andrews AJ (2019) Two factor authentication: Asf1 mediates crosstalk between H3 K14 and K56 acetylation. Nucleic Acids Res 47, 7380–7391. 31194870 10.1093/nar/gkz508 PMC6698667

[131] Fu I , Geacintov NE and Broyde S (2021) Molecular dynamics simulations reveal how H3K56 acetylation impacts nucleosome structure to promote DNA exposure for lesion sensing. DNA Repair 107, 103201. 34399316 10.1016/j.dnarep.2021.103201 PMC8526387

[132] Pollina EA , Gilliam DT , Landau AT , Lin C , Pajarillo N , Davis CP , Harmin DA , Yap E‐L , Vogel IR , Griffith EC *et al*. (2023) A NPAS4‐NuA4 complex couples synaptic activity to DNA repair. Nature 614, 732–741. 36792830 10.1038/s41586-023-05711-7 PMC9946837

[133] Chen C‐C , Carson JJ , Feser J , Tamburini B , Zabaronick S , Linger J and Tyler JK (2008) Acetylated lysine 56 on histone H3 drives chromatin assembly after repair and signals for the completion of repair. Cell 134, 231–243. 18662539 10.1016/j.cell.2008.06.035 PMC2610811

[134] Tamburini BA and Tyler JK (2005) Localized histone acetylation and deacetylation triggered by the homologous recombination pathway of double‐strand DNA repair. Mol Cell Biol 25, 4903–4913. 15923609 10.1128/MCB.25.12.4903-4913.2005 PMC1140608

[135] Li Q , Zhou H , Wurtele H , Davies B , Horazdovsky B , Verreault A and Zhang Z (2008) Acetylation of histone H3 lysine 56 regulates replication‐coupled nucleosome assembly. Cell 134, 244–255. 18662540 10.1016/j.cell.2008.06.018 PMC2597342

[136] Jiang X , Xu Y and Price BD (2010) Acetylation of H2AX on lysine 36 plays a key role in the DNA double‐strand break repair pathway. FEBS Lett 584, 2926–2930. 20488183 10.1016/j.febslet.2010.05.017 PMC2887596

[137] White DE , Rafalska‐Metcalf IU , Ivanov AV , Corsinotti A , Peng H , Lee SC , Trono D , Janicki SM and Rauscher FJ (2012) The ATM substrate KAP1 controls DNA repair in heterochromatin: regulation by HP1 proteins and serine 473/824 phosphorylation. Mol Cancer Res 10, 401–414. 22205726 10.1158/1541-7786.MCR-11-0134 PMC4894472

[138] Meyer B , Fabbrizi MR , Raj S , Zobel CL , Hallahan DE and Sharma GG (2016) Histone H3 lysine 9 acetylation obstructs ATM activation and promotes ionizing radiation sensitivity in Normal stem cells. Stem Cell Reports 7, 1013–1022. 27974220 10.1016/j.stemcr.2016.11.004 PMC5161741

[139] Di Giorgio E , Dalla E , Tolotto V , D'Este F , Paluvai H , Ranzino L and Brancolini C (2024) HDAC4 influences the DNA damage response and counteracts senescence by assembling with HDAC1/HDAC2 to control H2BK120 acetylation and homology‐directed repair. Nucleic Acids Res 52, 8218–8240. 38874468 10.1093/nar/gkae501 PMC11317144

[140] Min S , Lee H‐S , Ji J‐H , Heo Y , Kim Y , Chae S , Choi YW , Kang H‐C , Nakanishi M and Cho H (2021) The chromatin remodeler RSF1 coordinates epigenetic marks for transcriptional repression and DSB repair. Nucleic Acids Res 49, 12268–12283. 34850117 10.1093/nar/gkab1093 PMC8643642

[141] Terzi N , Churchman LS , Vasiljeva L , Weissman J and Buratowski S (2011) H3K4 trimethylation by Set1 promotes efficient termination by the Nrd1‐Nab3‐Sen1 pathway. Mol Cell Biol 31, 3569–3583. 21709022 10.1128/MCB.05590-11 PMC3165552

[142] Espinosa JM , Verdun RE and Emerson BM (2003) p53 functions through stress‐ and promoter‐specific recruitment of transcription initiation components before and after DNA damage. Mol Cell 12, 1015–1027. 14580351 10.1016/s1097-2765(03)00359-9

[143] Kaeser MD and Iggo RD (2004) Promoter‐specific p53‐dependent histone acetylation following DNA damage. Oncogene 23, 4007–4013. 15007388 10.1038/sj.onc.1207536

[144] Faucher D and Wellinger RJ (2010) Methylated H3K4, a transcription‐associated histone modification, is involved in the DNA damage response pathway. PLoS Genet 6, e1001082. 20865123 10.1371/journal.pgen.1001082 PMC2928815

[145] Gong F , Chiu L‐Y , Cox B , Aymard F , Clouaire T , Leung JW , Cammarata M , Perez M , Agarwal P , Brodbelt JS *et al*. (2015) Screen identifies bromodomain protein ZMYND8 in chromatin recognition of transcription‐associated DNA damage that promotes homologous recombination. Genes Dev 29, 197–211. 25593309 10.1101/gad.252189.114 PMC4298138

[146] Toker L , Tran GT , Sundaresan J , Tysnes O‐B , Alves G , Haugarvoll K , Nido GS , Dölle C and Tzoulis C (2021) Genome‐wide histone acetylation analysis reveals altered transcriptional regulation in the Parkinson's disease brain. Mol Neurodegener 16, 31. 33947435 10.1186/s13024-021-00450-7 PMC8097820

[147] Matsushita N (2023) Dysregulated histone acetylation causes congenital diseases. Gene Reports 31, 101778.

[148] Miziak P , Baran M , Borkiewicz L , Trombik T and Stepulak A (2024) Acetylation of histone H3 in cancer progression and prognosis. Int J Mol Sci 25, 10982. 39456765 10.3390/ijms252010982 PMC11507103

[149] Judes G , Dubois L , Rifaï K , Idrissou M , Mishellany F , Pajon A , Besse S , Daures M , Degoul F , Bignon Y‐J *et al*. (2018) TIP60: an actor in acetylation of H3K4 and tumor development in breast cancer. Epigenomics 10, 1415–1430. 30324811 10.2217/epi-2018-0004

[150] Samoilova EM , Romanov SE , Chudakova DA and Laktionov PP (2024) Role of sirtuins in epigenetic regulation and aging control. Vavilovskii Zhurnal Genet Selektsii 28, 215–227. 38680178 10.18699/vjgb-24-26 PMC11043508

[151] Chen D , Bruno J , Easlon E , Lin S‐J , Cheng H‐L , Alt FW and Guarente L (2008) Tissue‐specific regulation of SIRT1 by calorie restriction. Genes Dev 22, 1753–1757. 18550784 10.1101/gad.1650608 PMC2492662

[152] Ren X‐G , Li W , Li W‐X and Yu W (2024) Mechanism of histone arginine methylation dynamic change in cellular stress. Int J Mol Sci 25, 7562. 39062806 10.3390/ijms25147562 PMC11277302

[153] Mushtaq A , Mir US , Hunt CR , Pandita S , Tantray WW , Bhat A , Pandita RK , Altaf M and Pandita TK (2021) Role of histone methylation in maintenance of genome integrity. Genes 12, 1000. 34209979 10.3390/genes12071000 PMC8307007

[154] Mentch SJ , Merhmohamadi M , Huang L , Liu X , Gupta D , Mattocks D , Gomez P , Ables G , Bamman MM , Thalacker‐Mercer AE *et al*. (2015) Histone methylation dynamics and gene regulation occur through the sensing of one‐carbon metabolism. Cell Metab 22, 861–873. 26411344 10.1016/j.cmet.2015.08.024 PMC4635069

[155] Bayo J , Tran TA , Wang L , Peña‐Llopis S , Das AK and Martinez ED (2018) Jumonji inhibitors overcome Radioresistance in cancer through changes in H3K4 methylation at double‐Strand breaks. Cell Rep 25, 1040–1050. 30355483 10.1016/j.celrep.2018.09.081 PMC6245670

[156] Gong F , Clouaire T , Aguirrebengoa M , Legube G and Miller KM (2017) Histone demethylase KDM5A regulates the ZMYND8–NuRD chromatin remodeler to promote DNA repair. J Cell Biol 216, 1959–1974. 28572115 10.1083/jcb.201611135 PMC5496618

[157] Nakata Y , Nagasawa S , Sera Y , Yamasaki N , Kanai A , Kobatake K , Ueda T , Koizumi M , Manabe I , Kaminuma O *et al*. (2024) PTIP epigenetically regulates DNA damage‐induced cell cycle arrest by upregulating PRDM1. Sci Rep 14, 17987. 39097652 10.1038/s41598-024-68295-w PMC11297997

[158] Wang S , Meyer DH and Schumacher B (2020) H3K4me2 regulates the recovery of protein biosynthesis and homeostasis following DNA damage. Nat Struct Mol Biol 27, 1165–1177. 33046905 10.1038/s41594-020-00513-1

[159] Mosammaparast N , Kim H , Laurent B , Zhao Y , Lim HJ , Majid MC , Dango S , Luo Y , Hempel K , Sowa ME *et al*. (2013) The histone demethylase LSD1/KDM1A promotes the DNA damage response. J Cell Biol 203, 457–470. 24217620 10.1083/jcb.201302092 PMC3824007

[160] Di Nisio E , Lupo G , Licursi V and Negri R (2021) The role of histone lysine methylation in the response of mammalian cells to ionizing radiation. Front Genet 12, 639602. 33859667 10.3389/fgene.2021.639602 PMC8042281

[161] Montavon T , Shukeir N , Erikson G , Engist B , Onishi‐Seebacher M , Ryan D , Musa Y , Mittler G , Meyer AG , Genoud C *et al*. (2021) Complete loss of H3K9 methylation dissolves mouse heterochromatin organization. Nat Commun 12, 4359. 34272378 10.1038/s41467-021-24532-8 PMC8285382

[162] Huynh MT , Sengupta B , Krajewski WA and Lee T‐H (2023) Effects of histone H2B Ubiquitylations and H3K79me3 on transcription elongation. ACS Chem Biol 18, 537–548. 36857155 10.1021/acschembio.2c00887 PMC10023449

[163] Ljungman M , Parks L , Hulbatte R and Bedi K (2019) The role of H3K79 methylation in transcription and the DNA damage response. Mutat Res – Rev Mutat Res 780, 48–54. 31395348 10.1016/j.mrrev.2017.11.001

[164] Jørgensen S , Eskildsen M , Fugger K , Hansen L , Larsen MSY , Kousholt AN , Syljuåsen RG , Trelle MB , Jensen ON , Helin K *et al*. (2011) SET8 is degraded via PCNA‐coupled CRL4(CDT2) ubiquitylation in S phase and after UV irradiation. J Cell Biol 192, 43–54. 21220508 10.1083/jcb.201009076 PMC3019552

[165] Pei H , Zhang L , Luo K , Qin Y , Chesi M , Fei F , Bergsagel PL , Wang L , You Z and Lou Z (2011) MMSET regulates histone H4K20 methylation and 53BP1 accumulation at DNA damage sites. Nature 470, 124–128. 21293379 10.1038/nature09658 PMC3064261

[166] Ayrapetov MK , Gursoy‐Yuzugullu O , Xu C , Xu Y and Price BD (2014) DNA double‐strand breaks promote methylation of histone H3 on lysine 9 and transient formation of repressive chromatin. Proc Natl Acad Sci 111, 9169–9174. 24927542 10.1073/pnas.1403565111 PMC4078803

[167] Lutze J , Wolfgeher D and Kron SJ (2021) Global epigenetic analysis reveals H3K27 Methylation as a mediator of double strand break repair. 2021.09.20.461136.

[168] Goodarzi AA , Noon AT , Deckbar D , Ziv Y , Shiloh Y , Löbrich M and Jeggo PA (2008) ATM signaling facilitates repair of DNA double‐strand breaks associated with heterochromatin. Mol Cell 31, 167–177. 18657500 10.1016/j.molcel.2008.05.017

[169] Kakarougkas A , Ismail A , Chambers AL , Riballo E , Herbert AD , Künzel J , Löbrich M , Jeggo PA and Downs JA (2014) Requirement for PBAF in transcriptional repression and repair at DNA breaks in actively transcribed regions of chromatin. Mol Cell 55, 723–732. 25066234 10.1016/j.molcel.2014.06.028 PMC4157577

[170] Du J , Johnson LM , Jacobsen SE and Patel DJ (2015) DNA methylation pathways and their crosstalk with histone methylation. Nat Rev Mol Cell Biol 16, 519–532. 26296162 10.1038/nrm4043 PMC4672940

[171] Li L and Wang Y (2017) Crosstalk between the H3K36me3 and H4K16ac histone epigenetic marks in DNA double‐strand break repair. J Biol Chem 292, 11951–11959. 28546430 10.1074/jbc.M117.788224 PMC5512086

[172] Edmunds JW , Mahadevan LC and Clayton AL (2008) Dynamic histone H3 methylation during gene induction: HYPB/Setd2 mediates all H3K36 trimethylation. EMBO J 27, 406–420. 18157086 10.1038/sj.emboj.7601967 PMC2168397

[173] Pfister SX , Ahrabi S , Zalmas L‐P , Sarkar S , Aymard F , Bachrati CZ , Helleday T , Legube G , La Thangue NB , Porter ACG *et al*. (2014) SETD2‐dependent histone H3K36 Trimethylation is required for homologous recombination repair and genome stability. Cell Rep 7, 2006–2018. 24931610 10.1016/j.celrep.2014.05.026 PMC4074340

[174] Lee MY , Lee J , Hyeon SJ , Cho H , Hwang YJ , Shin J‐Y , McKee AC , Kowall NW , Kim J‐I , Stein TD *et al*. (2020) Epigenome signatures landscaped by histone H3K9me3 are associated with the synaptic dysfunction in Alzheimer's disease. Aging Cell 19, e13153. 32419307 10.1111/acel.13153 PMC7294781

[175] Das P and Taube JH (2020) Regulating methylation at H3K27: a trick or treat for cancer cell plasticity. Cancers 12, 2792. 33003334 10.3390/cancers12102792 PMC7600873

[176] Cui K , Zang C , Roh T‐Y , Schones DE , Childs RW , Peng W and Zhao K (2009) Chromatin signatures in multipotent human hematopoietic stem cells indicate the fate of bivalent genes during differentiation. Cell Stem Cell 4, 80–93. 19128795 10.1016/j.stem.2008.11.011 PMC2785912

[177] Huang Z , Hu L , Liu Z and Wang S (2025) The functions and regulatory mechanisms of histone modifications in skeletal muscle development and disease. Int J Mol Sci 26, 3644. 40332229 10.3390/ijms26083644 PMC12027200

[178] Han S and Brunet A (2012) Histone methylation makes its mark on longevity. Trends Cell Biol 22, 42–49. 22177962 10.1016/j.tcb.2011.11.001 PMC3253950

[179] Mattiroli F and Penengo L (2021) Histone ubiquitination: an integrative signaling platform in genome stability. Trends Genet 37, 566–581. 33485674 10.1016/j.tig.2020.12.005

[180] Chen JJ , Stermer D and Tanny JC (2022) Decoding histone ubiquitylation. Front cell Dev Biol 10, 968398. 36105353 10.3389/fcell.2022.968398 PMC9464978

[181] Mandemaker IK , van Cuijk L , Janssens RC , Lans H , Bezstarosti K , Hoeijmakers JH , Demmers JA , Vermeulen W and Marteijn JA (2017) DNA damage‐induced histone H1 ubiquitylation is mediated by HUWE1 and stimulates the RNF8‐RNF168 pathway. Sci Rep 7, 15353. 29127375 10.1038/s41598-017-15194-y PMC5681673

[182] Walser F , Mulder MPC , Bragantini B , Burger S , Gubser T , Gatti M , Botuyan MV , Villa A , Altmeyer M , Neri D *et al*. (2020) Ubiquitin phosphorylation at Thr12 modulates the DNA damage response. Mol Cell 80, 423–436. 33022275 10.1016/j.molcel.2020.09.017 PMC7655664

[183] Stewart GS , Stankovic T , Byrd PJ , Wechsler T , Miller ES , Huissoon A , Drayson MT , West SC , Elledge SJ and Taylor AMR (2007) RIDDLE immunodeficiency syndrome is linked to defects in 53BP1‐mediated DNA damage signaling. Proc Natl Acad Sci USA 104, 16910–16915. 17940005 10.1073/pnas.0708408104 PMC2040433

[184] Densham RM , Garvin AJ , Stone HR , Strachan J , Baldock RA , Daza‐Martin M , Fletcher A , Blair‐Reid S , Beesley J , Johal B *et al*. (2016) Human BRCA1‐BARD1 ubiquitin ligase activity counteracts chromatin barriers to DNA resection. Nat Struct Mol Biol 23, 647–655. 27239795 10.1038/nsmb.3236 PMC6522385

[185] Hao S , Wang Y , Zhao Y , Gao W , Cui W , Li Y , Cui J , Liu Y , Lin L , Xu X *et al*. (2022) Dynamic switching of crotonylation to ubiquitination of H2A at lysine 119 attenuates transcription–replication conflicts caused by replication stress. Nucleic Acids Res 50, 9873–9892. 36062559 10.1093/nar/gkac734 PMC9508856

[186] Wang H , Zhai L , Xu J , Joo H‐Y , Jackson S , Erdjument‐Bromage H , Tempst P , Xiong Y and Zhang Y (2006) Histone H3 and H4 ubiquitylation by the CUL4‐DDB‐ROC1 ubiquitin ligase facilitates cellular response to DNA damage. Mol Cell 22, 383–394. 16678110 10.1016/j.molcel.2006.03.035

[187] Ng H‐M , Wei L , Lan L and Huen MSY (2016) The Lys63‐deubiquitylating enzyme BRCC36 limits DNA break processing and repair. J Biol Chem 291, 16197–16207. 27288411 10.1074/jbc.M116.731927 PMC4965568

[188] Barbour H , Daou S , Hendzel M and Affar EB (2020) Polycomb group‐mediated histone H2A monoubiquitination in epigenome regulation and nuclear processes. Nat Commun 11, 5947. 33230107 10.1038/s41467-020-19722-9 PMC7683540

[189] Ciapponi M , Karlukova E , Schkölziger S , Benda C and Müller J (2024) Structural basis of the histone ubiquitination read–write mechanism of RYBP–PRC1. Nat Struct Mol Biol 31, 1023–1027. 38528151 10.1038/s41594-024-01258-x PMC11257959

[190] Moyal L , Lerenthal Y , Gana‐Weisz M , Mass G , So S , Wang S‐Y , Eppink B , Chung YM , Shalev G , Shema E *et al*. (2011) Requirement of ATM‐dependent monoubiquitylation of histone H2B for timely repair of DNA double‐strand breaks. Mol Cell 41, 529–542. 21362549 10.1016/j.molcel.2011.02.015 PMC3397146

[191] Oss‐Ronen L , Sarusi T and Cohen I (2022) Histone mono‐ubiquitination in transcriptional regulation and its mark on life: emerging roles in tissue development and disease. Cells 11, 2404. 35954248 10.3390/cells11152404 PMC9368181

[192] Nguyen AT and Zhang Y (2011) The diverse functions of Dot1 and H3K79 methylation. Genes Dev 25, 1345–1358. 21724828 10.1101/gad.2057811 PMC3134078

[193] Luo A , Kong J , Chen J , Xiao X , Lan J , Li X , Liu C , Wang P‐Y , Li G , Li W *et al*. (2023) H2B ubiquitination recruits FACT to maintain a stable altered nucleosome state for transcriptional activation. Nat Commun 14, 741. 36765085 10.1038/s41467-023-36467-3 PMC9918737

[194] Lee J‐S , Shukla A , Schneider J , Swanson SK , Washburn MP , Florens L , Bhaumik SR and Shilatifard A (2007) Histone crosstalk between H2B Monoubiquitination and H3 methylation mediated by COMPASS. Cell 131, 1084–1096. 18083099 10.1016/j.cell.2007.09.046

[195] Meas R and Mao P (2015) Histone ubiquitylation and its roles in transcription and DNA damage response. DNA Repair 36, 36–42. 26422137 10.1016/j.dnarep.2015.09.016 PMC4688114

[196] Mattiroli F , Vissers JHA , van Dijk WJ , Ikpa P , Citterio E , Vermeulen W , Marteijn JA and Sixma TK (2012) RNF168 ubiquitinates K13‐15 on H2A/H2AX to drive DNA damage signaling. Cell 150, 1182–1195. 22980979 10.1016/j.cell.2012.08.005

[197] Guo Y , Chomiak AA , Hong Y , Lowe CC , Kopsidas CA , Chan W‐C , Andrade J , Pan H , Zhou X , Monuki ES *et al*. (2022) Histone H2A ubiquitination resulting from Brap loss of function connects multiple aging hallmarks and accelerates neurodegeneration. iScience 25, 104519. 35754718 10.1016/j.isci.2022.104519 PMC9213774

[198] Yang L , Ma Z , Wang H , Niu K , Cao Y , Sun L , Geng Y , Yang B , Gao F , Chen Z *et al*. (2019) Ubiquitylome study identifies increased histone 2A ubiquitylation as an evolutionarily conserved aging biomarker. Nat Commun 10, 2191. 31113955 10.1038/s41467-019-10136-w PMC6529468

[199] Smerdon MJ (1991) DNA repair and the role of chromatin structure. Curr Opin Cell Biol 3, 422–428. 1892653 10.1016/0955-0674(91)90069-b

[200] Vineis P , Chatziioannou A , Cunliffe VT , Flanagan JM , Hanson M , Kirsch‐Volders M and Kyrtopoulos S (2017) Epigenetic memory in response to environmental stressors. FASEB J 31, 2241–2251. 28280003 10.1096/fj.201601059RR

